# Genomic Analysis of *Caldithrix abyssi*, the Thermophilic Anaerobic Bacterium of the Novel Bacterial Phylum *Calditrichaeota*

**DOI:** 10.3389/fmicb.2017.00195

**Published:** 2017-02-20

**Authors:** Ilya V. Kublanov, Olga M. Sigalova, Sergey N. Gavrilov, Alexander V. Lebedinsky, Christian Rinke, Olga Kovaleva, Nikolai A. Chernyh, Natalia Ivanova, Chris Daum, T.B.K. Reddy, Hans-Peter Klenk, Stefan Spring, Markus Göker, Oleg N. Reva, Margarita L. Miroshnichenko, Nikos C. Kyrpides, Tanja Woyke, Mikhail S. Gelfand, Elizaveta A. Bonch-Osmolovskaya

**Affiliations:** ^1^Winogradsky Institute of Microbiology, Research Center of Biotechnology, Russian Academy of SciencesMoscow, Russia; ^2^A.A.Kharkevich Institute for Information Transmission Problems, Russian Academy of SciencesMoscow, Russia; ^3^Australian Centre for Ecogenomics, School of Chemistry and Molecular Biosciences, University of Queensland, St LuciaQLD, Australia; ^4^DOE Joint Genome Institute, Walnut CreekCA, USA; ^5^School of Biology, Newcastle UniversityNewcastle upon Tyne, UK; ^6^Leibniz Institute DSMZ – German Collection of Microorganisms and Cell CulturesBraunschweig, Germany; ^7^Center for Bioinformatics and Computational Biology, Department of Biochemistry, University of PretoriaPretoria, South Africa; ^8^Biological Data Management and Technology Center, Lawrence Berkeley National Laboratory, BerkeleyCA, USA; ^9^Department of Bioengineering and Bioinformatics, M.V. Lomonosov Moscow State UniversityMoscow, Russia; ^10^Skolkovo Institute of Science and TechnologyMoscow, Russia; ^11^Faculty of Computer Science, National Research University – Higher School of EconomicsMoscow, Russia

**Keywords:** bacterial evolution, phylogenomics, taxonomy, phylum, *Caldithrix*, genomic analysis, sequencing

## Abstract

The genome of *Caldithrix abyssi*, the first cultivated representative of a phylum-level bacterial lineage, was sequenced within the framework of Genomic Encyclopedia of Bacteria and Archaea (GEBA) project. The genomic analysis revealed mechanisms allowing this anaerobic bacterium to ferment peptides or to implement nitrate reduction with acetate or molecular hydrogen as electron donors. The genome encoded five different [NiFe]- and [FeFe]-hydrogenases, one of which, group 1 [NiFe]-hydrogenase, is presumably involved in lithoheterotrophic growth, three other produce H_2_ during fermentation, and one is apparently bidirectional. The ability to reduce nitrate is determined by a nitrate reductase of the Nap family, while nitrite reduction to ammonia is presumably catalyzed by an octaheme cytochrome *c* nitrite reductase εHao. The genome contained genes of respiratory polysulfide/thiosulfate reductase, however, elemental sulfur and thiosulfate were not used as the electron acceptors for anaerobic respiration with acetate or H_2_, probably due to the lack of the gene of the maturation protein. Nevertheless, elemental sulfur and thiosulfate stimulated growth on fermentable substrates (peptides), being reduced to sulfide, most probably through the action of the cytoplasmic sulfide dehydrogenase and/or NAD(P)-dependent [NiFe]-hydrogenase (sulfhydrogenase) encoded by the genome. Surprisingly, the genome of this anaerobic microorganism encoded all genes for cytochrome *c* oxidase, however, its maturation machinery seems to be non-operational due to genomic rearrangements of supplementary genes. Despite the fact that sugars were not among the substrates reported when *C. abyssi* was first described, our genomic analysis revealed multiple genes of glycoside hydrolases, and some of them were predicted to be secreted. This finding aided in bringing out four carbohydrates that supported the growth of *C. abyssi*: starch, cellobiose, glucomannan and xyloglucan. The genomic analysis demonstrated the ability of *C. abyssi* to synthesize nucleotides and most amino acids and vitamins. Finally, the genomic sequence allowed us to perform a phylogenomic analysis, based on 38 protein sequences, which confirmed the deep branching of this lineage and justified the proposal of a novel phylum *Calditrichaeota*.

## Introduction

Bacterial and archaeal phyla represent the second highest divisions following domains. Although not yet covered by the International Code of Nomenclature of Prokaryotes (Oren et al., 2015), they reflect the early steps of evolution, which can be reconstructed by comparative analysis of their representatives.

The development and extensive application of molecular microbiology techniques over past decades revealed that cultivated microorganisms comprise at most 1% of all existing microorganisms on Earth (Amann et al., 1995). Our analysis of the SSU 123 ARB-Silva (Quast et al., 2013) revealed that only 0.45% of the SSU rRNA sequences in this database belong to cultivated strains (Kublanov et al., unpublished). Due to cultivation difficulties, the percentage of cultivated extremophilic microorganisms is even lower than that of mesophilic and neutrophilic ones (Kristjánsson and Hreggvidsson, 1995). According to the SSU 123 ARB-Silva database, 90% of the cultivated strains are distributed among four bacterial phyla: *Proteobacteria, Actinobacteria, Firmicutes*, and *Bacteroidetes*. The remaining phyla include few or even a single cultivated representative. Moreover, despite much effort directed toward the isolation of cultivated representatives of known candidate divisions (phyla without any cultivated representatives), many divisions (estimated as 35 to 50; McDonald et al., 2012; Rinke et al., 2013; Hedlund et al., 2015) still have no such representatives. Their metabolism has in many cases been reconstructed by various molecular techniques, mainly based on metagenomics and single cell genomics (Hedlund et al., 2015), however, not all of them have sufficient sequence representation and thus information on coding potential. While these widely applied approaches are clearly useful, efforts focused on isolation and cultivation of the first representatives of deep phylogenetic lineages of bacteria and archaea are highly important.

Bacteria of the genus *Caldithrix* represent a deep phylogenetic lineage, which has been first identified in a 16S rRNA clone library obtained from shallow-water hydrothermal sediments in Aegean Sea (Sievert et al., 2000). Independently, the first cultivated representative of this group, *Caldithrix abyssi*, was isolated from a Mid-Atlantic Ridge deep-sea hydrothermal chimney sample (Miroshnichenko et al., 2003). A 16S rRNA-based phylogenetic analysis placed *C. abyssi* as a deep phylum-level branch. Phenotypically, *C. abyssi* is a bacterium with rod-shaped cells sometimes forming large bubbles, moderately thermophilic, neutrophilic and obligately anaerobic. Its metabolism is versatile and indicates its ability to participate in various microbial processes taking place in deep-sea hydrothermal environments. According to the original description, *C. abyssi* can grow lithoheterotrophically with molecular hydrogen as the electron donor and nitrate as the electron acceptor, reducing the latter to ammonium. It can also perform anaerobic oxidation of acetate coupled with nitrate reduction to ammonium. *C. abyssi* also grows by fermentation of peptides (Miroshnichenko et al., 2003; Fedosov et al., 2006). It has been suggested that such a flexible metabolism helps *C. abyssi* to adapt to changing conditions of the deep-sea hydrothermal environment.

At the beginning of this study, *C. abyssi* was the only cultivated representative of this deep phylum-level lineage. Then another representative of the genus, *C. palaeochoriensis*, was isolated from a Palaeochori Bay (Aegean Sea) hot vent (Miroshnichenko et al., 2010). The goal of the present study was to get insight into specific features of the deep phylogenetic lineage represented by the genus *Caldithrix.* To this end, we performed a comprehensive analysis of the *de novo* sequenced genome of *C. abyssi* strain LF13, which allowed us to predict its physiology in more detail and led us to experimentally validate some of these genomic predictions. Phylogenomic analysis supported the deep, phylum-level position within the tree of life, forming a basis for the proposal of a novel phylum *Calditrichaeota*.

## Materials and Methods

### Cultivation of *C. abyssi* Strain LF13 and Growth Substrates/Electron Acceptors Verification

For the verification of novel genomic data, *C. abyssi* strain LF13 was cultivated on the same basal medium (Miroshnichenko et al., 2003) supplemented with electron donors and, if necessary, acceptors that were either analyzed previously in the initial study (Miroshnichenko et al., 2003) but needed re-evaluation, or were earlier untested compounds prompted by genomic analysis. The fermentable substrates tested were maltose, pullulan, dextran, dextrin starch, trehalose, cellobiose, cellulose, lactose, sucrose, chitin, chitosan, pectin, agarose, alginate, xyloglucan, and glucomannan (Supplementary Table 2). The following compounds were tested as possible electron donors with elemental sulfur (10 g l^-1^) and thiosulfate (20 mM) as electron acceptors: hydrogen (1:1 with N_2_ in 8 ml of the gas phase of an 18-ml tube), acetate (17 mM) and peptone (2 g l^-1^). The ability of *C. abyssi* to grow aerobically was tested with acetate and peptone as the electron donors at the concentrations of oxygen in the gas phase varying from 0.1 to 15%.

### Genome Sequencing and Assembly

*Caldithrix abyssi* genomic DNA was obtained from *C. abyssi* LF13 cells grown on a basal medium (Miroshnichenko et al., 2008) supplemented with soy bean (2 g l^-1^). The DNA was isolated and purified following the procedure described by Chernyh et al. (2015).

*Caldithrix abyssi* LF13 (DSM 13497^T^) genome was sequenced within the framework of the Genomic Encyclopedia of *Bacteria* and *Archaea* (GEBA) project (Wu et al., 2009; Klenk and Göker, 2010), which was based on phylogeny-driven strain selection (Göker and Klenk, 2013). The genome sequence was generated at the DOE Joint genome Institute (JGI) using a combination of Illumina (Bennett, 2004) and 454 technologies (Margulies et al., 2005). All general aspects of library construction and sequencing performed at the JGI can be found at http://www.jgi.doe.gov/. The initial draft assembly contained 313 contigs in 1 scaffold. The 454 Titanium standard data and the 454 paired end data were assembled together with Newbler, version 2.3-PreRelease-6/30/2009. The Newbler consensus sequences were computationally shredded into 2 kb overlapping fake reads (shreds). Illumina sequencing data was assembled with VELVET, version 1.0.13 (Zerbino and Birney, 2008), and the consensus sequences were computationally shredded into 1.5 kb overlapping fake reads (shreds). We integrated the 454 Newbler consensus shreds, the Illumina VELVET consensus shreds and the read pairs in the 454 paired end library using parallel phrap, version 1.080812 (High Performance Software, LLC). The software Consed (Ewing and Green, 1998; Ewing et al., 1998; Gordon et al., 1998) was used in the following finishing process. Illumina data was used to correct potential base errors and increase consensus quality using the software Polisher developed at JGI (Alla Lapidus, unpublished). Possible mis-assemblies were corrected using gapResolution (Cliff Han, unpublished), Dupfinisher (Han and Chain, 2006), or sequencing cloned bridging PCR fragments with subcloning. Gaps between contigs were closed by editing in Consed, by PCR and by Bubble PCR (Cheng, unpublished) primer walks. The estimated genome size was 5 Mb and the final assembly was based on 152.8 Mb of 454 draft data which provides an average 30.6x coverage of the genome and 407.7 Mb of Illumina draft data which provides an average 81.5x coverage of the genome.

### Structural and Functional Annotation

The origin site was predicted using the OriFinder software (Gao and Zhang, 2008). The genomic DNA G+C content was calculated *in silico* using the complete genome sequence.

Initial functional annotations of *C. abyssi* genes were obtained from the RAST Server (Aziz et al., 2008; Overbeek et al., 2014) and Integrated Microbial Genomics (IMG; Markowitz et al., 2014) pipelines. Assignment of conserved protein families domains was performed using hmmscan (Finn et al., 2015). Initial reconstruction of metabolic pathways was based on mapping to the KEGG (Kanehisa et al., 2015) and MetaCyc (Caspi et al., 2014) databases. Where applicable, missing steps were reconstructed based on the analysis of functional domains, genome context and presence of specific regulatory sequences [transcription factor (TF) binding sites or riboswitches]. Some of the approaches used are described by Toshchakov et al. (2015).

Horizontally transferred genomic islands in the chromosomal sequence of *C. abyssi* LF13 (DSM 13497) were identified by SeqWord Genomic Island Sniffer (SWGIS; Bezuidt et al., 2009) and by IslandViewer (Langille and Brinkman, 2009). Genomic islands were searched for sequences with similar oligonucleotide usage patterns through GOHTAM database (Ménigaud et al., 2012) and through Pre_GI database (Pierneef et al., 2015). Distances between oligonucleotide patterns of genomic islands were calculated as described by Ganesan et al. (2008).

DNA-binding proteins were identified with the InterProScan 5.18-57.0 search software (Hunter et al., 2009) as those having Pfam domains corresponding to the GO:0003677 (DNA binding) and GO:0006355 (regulation of transcription, DNA-templated). The resulting list was further subdivided into TFs and Other DNA-binding proteins (ODFs, including transposases, restriction-modification systems, DNA replication and repair machinery) by manual curation taking into account functional annotations from the RAST and IMG pipelines, Pfam domains of TFs and BLAST (Altschul et al., 1997) search against NCBI nucleotide or protein non-redundant database. As a result, 197 proteins with DNA-binding domains were identified in the *C. abyssi* genome, 85 of them were annotated as TFs.

Motifs for known TF binding sites were taken from the RegPrecise database (Novichkov et al., 2013). Sequence motifs search was performed with the MEME suite (Bailey et al., 2009) and GenomeExplorer software (Mironov et al., 2000). The search for riboswitches was performed by scanning HMM profiles from the Rfam database (Nawrocki et al., 2015) with the Infernal program (Nawrocki and Eddy, 2013). Analysis of overrepresented palindromes was performed with *ad hoc* R scripts.

Chromosomal cassettes comprising the genes of respiratory complex IV were retrieved using the IMG pipeline procedure of “Chromosomal cassette search by the COG terms” [COG0843, COG1622, COG1845, COG5605, COG1612, and COG0109, corresponding to CoxI-IV cytochrome oxidase subunits, heme *a* synthase (Cox15), and polyprenyltransferase, respectively]. The search was performed among all finished, permanent draft and draft genomes of Bacteria in the IMG database on December 27, 2015. The result was filtered for the terms “Culture Type = Isolate” and “Cultured = Yes” from the IMG Project information pages to extract data related to cultivated organisms only. The procedure retrieved 104 chromosomal cassettes, containing a complete set of the inquired COGs, in 104 cultivated bacterial strains. The analysis of the cassettes revealed peculiarities in the positions of genes encoding heme-processing enzymes in *C. abyssi*. Additional search for chromosomal cassettes was performed using *cox15* gene of *C. abyssi*, encoding heme *a* synthase, as a query with standard parameters in IMG pipeline. This search retrieved five more chromosomal cassettes containing all the COGs of cytochrome oxidase subunits mentioned above except the one for CoxIV subunit. Further manual curation revealed the presence of *coxIV* genes in these five cassettes which were not properly annotated in the database. So, a total of 109 chromosomal cassettes of cytochrome oxidases were used for further analysis. Analysis of metabolic information on the strains possessing the retrieved chromosomal cassettes was performed manually by analysis of IMG project information under the term “Oxygen requirement,” as well as information from NCBI/BioSample database, culture collections, and publications in International Journal of Systematic and Evolutionary Microbiology for validly published species.

### Phylogenetic Analysis

Genome assemblies of up to 2506 bacterial and archaeal taxa were scanned for homologs of a set of 38 universally conserved single-copy proteins present in Bacteria and Archaea (Rinke et al., 2013). The assemblies were translated into all six reading frames and marker genes were detected and aligned with hmmsearch and hmmalign included in the HMMER3 package (Eddy, 2011) using HMM profiles obtained from phylosift^1^. Extracted marker protein sequences were used to build concatenated alignments of 38 markers per genome.

The phylogenetic inference method used was the maximum likelihood and minimum evolution based implementation FastTree2 (Price et al., 2010) with a CAT model approximation with 20 rate categories and the Jones-Taylor-Thorton (JJT) substitution model. The resulting trees were imported into ARB (Ludwig et al., 2004), grouped into clades, exported as newick trees and beautified with iTOL (Letunic and Bork, 2011).

120 bacteria-specific conserved proteins phylogenetic analysis protocol is described in the Supplemental Materials.

## Results

### General Features of the *C. abyssi* Genome

The complete 4.98 Mb genome sequence of strain LF13 was deposited in IMG/GOLD (Ga0101742) and Genbank (CP018099). There were totally 4267 genes, among which 4219 were protein-coding, 3 were rRNA-coding and 45 were tRNA-coding genes. The genomic DNA G+C content was 45.1 mol%. This deviates by more than 1% from the G+C content value of 42.5 mol% indicated in the original species description (Miroshnichenko et al., 2003) and thus calls for an according emendation (Meier-Kolthoff et al., 2014).

### Energy Substrates: Primary and Central Metabolism

According to the original publication (Miroshnichenko et al., 2003), the energy substrates utilized by *C. abyssi* include molecular hydrogen, acetate, pyruvate, and hydrolyzed proteinaceous substrates, such as yeast, soy bean and beef extracts, peptone and casamino acids. No growth was detected with starch, cellobiose, dextrin, sucrose, glucose, galactose, xylose, maltose, ethanol, methanol, mannitol, propionate, butyrate or lactate.

In agreement with the previously reported abilities of *C. abyssi* to grow lithoheterotrophically with molecular hydrogen, as well as to produce hydrogen in the course of fermentation, five hydrogenases were found to be encoded in its genome. Below all these enzymes are considered in order to distinguish hydrogenases involved in hydrogen uptake from those involved in hydrogen production.

The catalytic subunits of the three encoded [NiFe]-hydrogenases are Cabys_2298, Cabys_3229, and Cabys_1914. All three exhibit the two characteristic CxxC Ni-binding motifs (Vignais and Billoud, 2007). Cabys_2299-2297 is an uptake hydrogenase of group 1 of [NiFe]-hydrogenases (Vignais and Billoud, 2007), which follows from the fact that one of its subunits (Cabys_2297) is cytochrome *b* and from the presence of group-characteristic L1 and L2 motifs in the catalytic subunit. Cabys_3226-3229 is a bidirectional NADP-dependent hydrogenase (group 3b) as judged from percent identity with biochemically ascertained homologs, the order of subunit-encoding genes, the presence of group-characteristic L1 and L2 motifs in the amino acid sequence of the catalytic subunit, and the lack of transmembrane helices in all four subunits. Cabys_1916-1914 is most probably a ferredoxin-dependent group 3c [NiFe]-hydrogenase. This assignment is confirmed by the phylogeny of the catalytic subunit (data not shown), and by the presence of group-characteristic L1 and L2 motifs; this hydrogenase is soluble, as judged from lack of transmembrane helices in its subunits, and probably ferredoxin-dependent. All three gene clusters encoding [NiFe]-hydrogenases contain genes encoding hydrogenase maturation proteases.

The *C. abyssi* genome contains genes for two [FeFe]-hydrogenases. Their catalytic (H_2_-producing) subunits Cabys_3678 and Cabys_1517 exhibit three characteristic sequence signatures P1, P2, and P3 (Vignais and Billoud, 2007). The Cabys_3676-3678 hydrogenase subunits are 54-56-37% identical to subunits Moth_1719-1717, respectively, of the [FeFe]-hydrogenase of *Moorella thermoacetica*, biochemically characterized as a reversible electron-bifurcating ferredoxin- and NAD-dependent [FeFe]-hydrogenase (Wang et al., 2013). However, while Moth_1719-1717 is most probably a distinct operon, the *C. abyssi* gene cluster additionally includes an upstream gene for the transcriptional regulator Rex (Cabys_3675). Rex proteins are known to sense the NADH/NAD^+^ ratio and, at its low values, to bind to specific target site(s), repressing transcription of genes involved in NADH oxidation (Ravcheev et al., 2012). Both DNA-binding and NAD-sensing domains are broadly conserved in Rex orthologs identified in several bacterial phyla, and the identified DNA-binding motifs also shows significant conservation, the generalized consensus being TTGTGAANNNNTTCACAA (Ravcheev et al., 2012). In the *C. abyssi* genome, 38 bp upstream of the Rex-encoding gene Cabys_3675, we found a candidate Rex binding site with two deviations from the above consensus (cTGTGAATCAATTCACAt). Thus, Cabys_3675-3679 is likely an operon whose transcription is repressed at a low NADH/NAD^+^ ratio. This ratio is expected to be low in the presence of appropriate electron acceptors (e.g., nitrate). Thus, despite its similarity to the reversible hydrogenase of *Moorella thermoacetica* Moth_1719-1717, Cabys_3675-3679 is not involved in H_2_ uptake, but oxidizes NADH and ferredoxin simultaneously in a synergistic fashion to produce H_2_. One more [FeFe]-hydrogenase is Cabys_1517, presumably a monomeric ferredoxin-dependent [FeFe]-hydrogenase, also involved in H_2_ production.

Thus, the set of *C. abyssi* hydrogenases that may be operative during lithoheterotrophic growth with H_2_ at the expense of nitrate reduction to ammonium includes the uptake [NiFe]-hydrogenase Cabys_2299-2297 and the bidirectional NAD(P)-dependent [NiFe]-hydrogenase Cabys_3226-3229.

Several homologs of known acetate transporters of the TCDB subfamily 2.A.21.7.2 (Saier et al., 2009), belonging to the solute:sodium symporter (SSS, 2.A.21) family, were found but their exact specificity could not be confidently predicted. Acetate activation occurs via an irreversible ATP-dependent two-step reaction, catalyzed by acetyl-CoA synthetase (ACS) Cabys_0302. Besides, the pathway catalyzed by acetate kinase Cabys_3462 and phosphate acetyltransferases Cabys_0020 and Cabys_0343 (PTA-ACKA), usually involved in the acetate production in the course of fermentation, might proceed in the reverse direction when acetate is present at high concentrations (Wolfe, 2005).

The ability of *C. abyssi* to grow on complex proteinaceous substrates, such as beef extract, soy bean, peptone and yeast extract, is in agreement with the presence of more than 150 genes encoding peptidases, both extracelluar and intracelluar.

The *C. abyssi* genome contains genes for more than 60 glycosidases (GHs), five polysaccharide lyases (PLs), eight carbohydrate esterases (CEs; Supplementary Table 1) and a large number of MFS (2.A.1), SSS (2.A.21) and ABC transporters (3.A.1), capable of transporting various compounds including sugars. More specific are the PTS (4.A) phoshorylation-coupled translocators of monosugars Cabys_3887-3890 and Cabys_0064-0067.

The subsequent growth experiments revealed the carbohydrates that were used by *C. abyssi* as substrates: starch, cellobiose, xyloglucan, and glucomannan (Supplementary Table 2).

**FIGURE 1:**
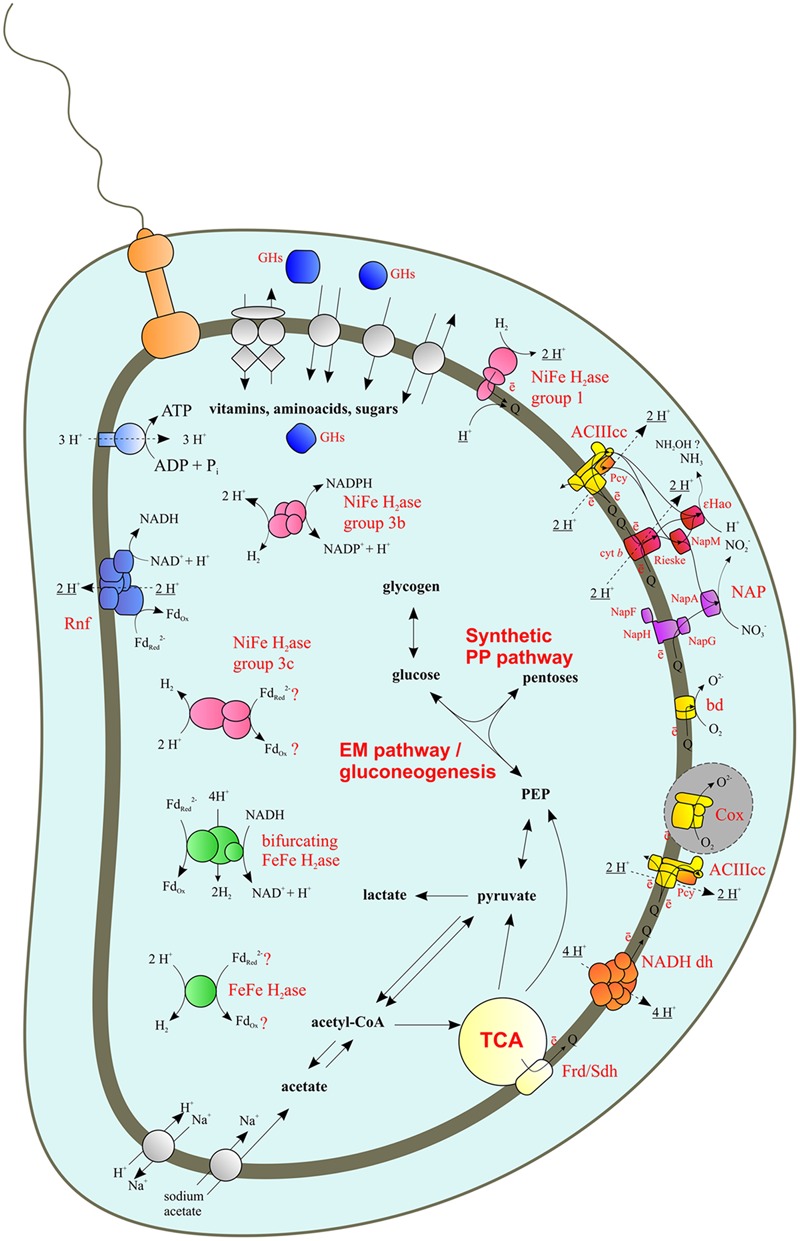
**Reconstructed energy metabolism of *Caldithrix abyssi* LF13.** NADH dh, NADH dehydrogenase (Complex I); Frd/Sdh, fumarate reductase/succinate dehydrogenase (Complex II); ACIIIcc, alternative complex III; bd, cytochrome bd oxidase; Cox, terminal heme-copper oxygen reductase; 𝜀Hao, nitrite reductase 𝜀Hao; NAP, nitrate reductase complex of Nap family; NapA, periplasmic molybdopterin-containing nitrate reductase; NapGH, quinol-oxidizing complex of FeS proteins; NapM, putative periplasmic electron carrier (tetraheme *c*-type cytochrome); NapF, auxiliary FeS protein facilitating NapA maturation and transportation to the periplasm; Pcy, plastocyanin; Rieske and cyt *b* - Rieske and cytochrome *b* subunits of the putative quinol-oxidizing complex, donating electrons to NapM and/or eHao; H_2_ase, hydrogenase; GHs, glycosidases; Gray area, surrounded by dotted line: all genes were found, however, the Cox biosynthesis machinery seems to be defective, thereby, *C. abyssi* does not respire oxygen. Electrogenic protons are underlined.

The genome of *C. abyssi* contains all genes of the Embden-Meyerhof (EM) and gluconeogenesis pathways of hexoses utilization and synthesis (**Figure 1**, Supplementary Table 3). The key enzymes of the Entner-Doudoroff (ED) pathway are absent. All genes coding for the enzymes of the synthetic part of the pentose-phosphate (PP) pathway were found; however, the genes encoding enzymes of the oxidative part (glucose-6-phosphate dehydrogenase and 6-phosphogluconolactonase) were not found (Supplementary Table 4). All genes of the oxidative tricarboxylic acid (TCA) cycle were found (**Figure 1**, Supplementary Table 5).

The TCA intermediates malate and oxaloacetate could be decarboxylated to pyruvate and phosphoenol pyruvate (PEP), respectively, by NADP^+^-dependent malic enzyme Cabys_2313 and ATP PEP carboxylase Cabys_0267.

### Terminal Oxidation Reactions and Oxidative Phosphorylation

Apart from fermentation of peptides and carbohydrates, *C. abyssi* is able to grow by nitrate reduction with acetate or hydrogen as substrates. Neither sulfur, thiosulfate, sulfate nor oxygen respiration supported its growth, as it was shown by Miroshnichenko et al. (2003) and confirmed here. With peptone as a substrate, sulfur and thiosulfate slightly stimulated fermentative growth and were reduced to sulfide.

In the course of carbohydrate or amino acid fermentation, NADH, NADPH and reduced ferredoxin are produced. Among the enzymes producing reduced ferredoxin there is e.g., pyruvate:ferredoxin oxidoreductase Cabys_0262, which was highly active during yeast extract fermentation, as shown by Fedosov et al. (2006). This enzyme may be involved both in carbohydrate and amino acid fermentations. Several *C. abyssi* hydrogenases take part in the disposal of the reducing equivalents. Neither of the *C. abyssi* hydrogenases is an energy-converting hydrogenase (group 4 [NiFe]-hydrogenase according to Vignais and Billoud (2007)), given low identity of their catalytic subunits to those of known energy-converting hydrogenases and lack of membrane-bound subunits (except the cytochrome *b* subunit of the uptake hydrogenase). Thus, *C. abyssi* cannot directly convert the energy of the reaction Fd_red_ → H_2_ to the energy of the transmembrane potential. The H_2_ production from reduced ferredoxin may be catalyzed by the presumably ferredoxin-dependent [NiFe]- and [FeFe]-hydrogenases Cabys_1916-1914 and Cabys_1517 (see the description of hydrogenases in the previous section). A pathway more favorable in terms of energy gain would be to channel the electrons of reduced ferredoxin to NAD via the ferredoxin:NAD-oxidoreductase Rnf, encoded in the *C. abyssi* genome by *rnfBCDGEA* (Cabys_3129-3134). This electron transfer is coupled to the generation of the transmembrane potential and thus to energy conservation (Biegel et al., 2011; Buckel and Thauer, 2013). The electrons from NADH, formed in the fermentation or due to the Rnf activity, can be used for the formation of reduced fermentation products other than hydrogen (such as propionate, reported to be among the products of yeast extract fermentation by Miroshnichenko et al. (2003), or for hydrogen production by NAD(P)-dependent bidirectional [NiFe]-hydrogenase Cabys_3226-3229 (at low H_2_ concentrations) or by electron-bifurcating [FeFe]-hydrogenase Cabys_3676-3678, which oxidizes NADH and ferredoxin simultaneously in a synergistic fashion and thus can produce H_2_ at its higher ambient concentrations.

The *C. abyssi* genome contains all essential genes of the respiratory electron transfer chain (**Figure 1**). Complex I is encoded by Cabys_4044-4056, a complete set of genes of the proton-translocating NADH-dehydrogenase *ndhAB(C/D)EFGHIJKLMN*. There is another putative NADH-dehydrogenase locus (Cabys_1521-1531) lacking genes for the NuoEFG subunits, which form the dehydrogenase domain involved in NADH binding and oxidation (Sazanov, 2007). Thus, this second *ndh* locus likely encodes an alternative membrane-bound proton-transporting complex accepting electrons from donors other than NADH (e.g., reduced ferredoxin).

Succinate dehydrogenase/fumarate reductase (Complex II) is encoded by Cabys_4073-4075. *C. abyssi* has no canonical cytochrome *bc_1_*-complex. Only two genes (Cabys_2558 and Cabys_2557) were found to encode proteins with Rieske and cytochrome *b* domains, similar to those of cytochrome *bc_1_*-complexes. However, both genes belong to the nitrate reductase locus *Nap* (see below), and no type 1 monoheme cytochrome *c* genes occur in close vicinity. Instead, an alternative complex III (ACIIIcc, Refojo et al., 2012) *actABCEED* was found: (Cabys_3114, 3113, 3112, 3111, 3110, and 3109, respectively, **Figure 1**).

**FIGURE 2:**
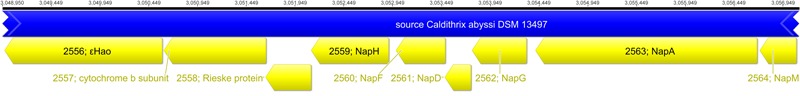
**The gene cluster encoding nitrate reductase Nap and putative nitrite reductase 𝜀Hao**.

*Caldithrix abyssi* is able to grow by nitrate reduction with molecular hydrogen or acetate as the electron donors (Miroshnichenko et al., 2003). The *nar* genes for membrane-bound intracellular nitrate reductase were not found, neither were nitrate transporters, indicating the absence of denitrification (Kraft et al., 2011). However, dissimilatory nitrate reduction can be mediated by dissimilatory periplasmic nitrate reductase Nap, encoded by Cabys_2564-2557 (**Figure 2**). While no genes for NrfAH or any other nitrite reductases were found, nitrite reduction can be performed by periplasmic *c*-type octaheme oxidoreductase 𝜀Hao, encoded by Cabys_2556, located adjacently to the *Nap* cluster. The *Nap* cluster is organized similarly to that described for the nitrate-reducing *Desulfovibrio desulfuricans* (Marietou et al., 2005): it lacks the *napB* gene but contains *napM* (Cabys_2564), encoding a tetraheme cytochrome *c*, which is considered to be the electron donor of the periplasmic catalytic subunit NapA (Cabys_2563). Notably, *C. abyssi* lacks NapC, the putative quinol-oxidizing electron donor of the NapM protein (Marietou et al., 2005; Simon and Klotz, 2013). However, the *C. abyssi*’s Nap cluster contains the genes of Rieske/cytochrome *b* complex (Cabys_2558 and Cabys_2557; **Figures 1** and **2**), which can oxidize quinols. Alternatively, the NapA nitrate reductase may accept electrons from the NapG iron-sulfur protein, which faces the periplasm and is a part of the quinol-oxidizing NapGH complex (Cabys_2562, Cabys_2559, **Figures 1** and **2**). In contrast to *D. desulfuricans* (Marietou et al., 2005), the *C. abyssi*’s NapG has a twin-arginin signal peptide, predicted by a TatP server (Bendtsen et al., 2005), suggesting its periplasmic localization. The nitrite reductase 𝜀Hao of *C. abyssi* can obtain electrons from quinols via the ACIIIcc complex or the Rieske/cytochrome *b* complex, encoded in the Nap cluster. In both cases, a tetraheme NapM is likely to serve as the main mediator of the electron transfer to the 𝜀Hao (**Figure 1**). The electron flow from ACIIIcc to NapM could be provided by a plastocyanin (Cabys_4150 and Cabys_4149), since not a single soluble monoheme cytochrome *c* protein was found to be encoded in the genome of *C. abyssi*.

Although according to Miroshnichenko et al. (2003) nitrate respiration was the only energy-gaining process in addition to fermentation, the genome analysis seemed to reveal a possibility of sulfur, thiosulfate, tetrathionate, and oxygen respiration. A polysulfide/thiosulfate reductase, whose catalytic subunit A is a member (Psr/Phs) of the molybdopterin superfamily (Duval et al., 2008), is encoded by Cabys_1269-1267 (ABC subunits, no homologs of the TorD maturation protein were encoded), and located close to two single-domain sulfur transferases genes Cabys_1264-1265. In addition, homologs of both subunits of the non-energy-conserving archaeal sulfide dehydrogenase SudBA (Ma and Adams, 1994) are encoded by Cabys_0800-0801. While no molybdopterin superfamily (Duval et al., 2008) tetrathionate reductases (Ttr) were found, a putative octaheme tetrathionate reductase (Otr) Cabys_2302, homologous to the one from *Shewanella* (Mowat et al., 2004), was identified. An uptake [NiFe]-hydrogenase Cabys_2299-2297 is encoded in close vicinity to this Otr, indicating possible tetrathionate reduction with H_2_ as the electron donor. However, subsequent growth experiments failed to support the predictions of sulfur/polysulfide/thiosulfate respiration (see next paragraph for consideration of the possibility of oxygen respiration). As for sulfate reduction, the necessary genes were not found.

*Caldithrix abyssi* possesses a gene cluster encoding major subunits of terminal heme *a*- and copper-containing cytochrome *c* oxidase CoxII, I, III, IV (Cabys_3106-3103), as well as a copper chaperone SCO1 involved in the maturation of the copper Cu(A) center in the CoxII subunit (Cabys_3107, Cabys_3101), heme *a* synthase interconverting hemes *a* and *o* (Cabys_3102), and polyprenyltransferase (heme *o* synthase, Cabys_3115), also involved in oxygen reductase maturation. However, the gene organization of oxygen reductase cluster in *C. abyssi* is unusual. Indeed, chromosomal cassette search in the IMG portal by COG terms corresponding to CoxI, CoxII, CoxIII, and CoxIV oxygen reductase subunits, heme *a* synthase (Cox15), and polyprenyltransferase (CtaB) retrieved 109 chromosomal cassettes (Supplementary Table 6) from all bacterial genomes in that database. Almost all these cassettes (106 of 109) exhibit a conserved gene organization (Supplementary Figure 1); moreover, an overwhelming majority (100 of 106) also contains a *sco1* gene at their 3′ end. Three atypical cassettes come from extremophiles (including *C. abyssi*). Most dissimilar to others is the oxygen reductase cassette predicted in *C. abyssi*, in which the set of core genes contains only one copy of *coxIII*, the genes of two major heme-processing enzymes Cox15 and CtaB are encoded separately from each other, and the gene *ctaB* of polyprenyltransferase is located well upstream of the core cluster of Cox and ACIIIcc complexes (Supplementary Figure 1). Apparently, such separation of the core heme-processing genes leads to the inability of *C. abyssi* to produce active cytochrome oxidase and respire oxygen. Yet, it is able to resist microaerophilic conditions, most probably due to the action of a catalase/peroxidase (Cabys_0410).

### Biosynthetic Processes

The *C. abyssi* genome contains candidate genes for complete biosynthesis pathways of nucleotides and most standard amino acids. Several pathways that are either incomplete or involve non-orthologous gene displacement, are discussed below. First, the initial step of the glutamate-to-arginine conversion is catalyzed by Cabys_1732, a newly characterized isoform of N-acetylglutamate synthase (ArgA, our unpublished results), encoded within the arginine locus *argAGCBEH* (Cabys_1732-1737). So far, the gene distribution is limited to the *Bacteroidetes* phylum and *C. abyssi.* Second, the standard pathway of proline biosynthesis from glutamate is incomplete; however, candidate genes for the ornithine-to-proline conversion are present in the genome (Cabys_3334 and Cabys_1741). Third, the 3-phospho-D-glycerate-to-serine pathway in *C. abyssi* lacks the step catalyzed by phosphoserine phosphatase (SerB), which converts phosphoserine to serine. However, this enzyme belongs to a large superfamily of haloacid dehalogenase (HAD)-like hydrolases (Kuznetsova et al., 2006), and, as multiple genes encoding proteins from this family have been found in the genome of *C. abyssi*, one of them could play the role of SerB. Moreover, serine could be synthesized from glycine, as all enzymes for the interconversions between serine and glycine, glycine and threonine, as well as for threonine synthesis from oxaloacetate are encoded in the genome. Finally, *C. abyssi* possesses genes for a rare type of lysine biosynthesis found in *Thermus thermophilus, Deinococcus radiodurans*, and several hyperthermophilic archaea (Kosuge and Hoshino, 1998; Nishida et al., 1999). Genes encoding proteins LysWXZYJ and Hcs of this pathway form one locus (Cabys_3146-3153). Genes encoding homologs of other proteins of the pathway are scattered over the genome (some of them occurring in the TCA cycle and arginine loci); therefore, further analysis is needed to establish their exact specificity.

Our analysis of *C. abyssi* genome revealed the ability of the bacterium to synthesize riboflavin (vitamin B2), pantothenate (vitamin B5), pyridoxal 5’-phosphate (PLP, vitamin B6 group), folate (vitamin B9) and its derivatives, heme, flavin adenine dinucleotide (FAD), flavin mononucleotide (FMN), coenzyme A (CoA), nicotinamide adenine dinucleotide (NAD), and nicotinamide adenine dinucleotide phosphate (NADH). In the folate biosynthesis pathway, dihydrofolate reductase gene *folA* is missing, its function being substituted for by dihydromonapterin reductase *folM* (Giladi et al., 2003). The biotin (vitamin B7) biosynthesis pathway is probably incomplete, and no homologs of known biotin transporters were found. The thiamine and cobalamin biosynthesis pathways are also incomplete, but candidate transporters have been identified (see Regulation).

*Caldithrix abyssi* has a fatty acid biosynthesis (*fab*) gene cluster (Cabys_3455-3460) with the gene content and order similar to those in *E. coli* (Rawlings and Cronan, 1992): phosphate acyltransferase (*plsX*), 3-ketoacyl-acyl carrier protein (ACP) synthase III (*fabH*), malonyl CoA-ACP transacylase (*fabD*), 3-ketoacyl-ACP reductase (*fabG*), ACP, and 3-ketoacyl-ACP synthase II (*fabF*) genes. Some *fab* genes (e.g., *fabF, fabG*, and *fabD*) were found in multiple copies. Close homologs of known *fabI* (enoyl-ACP reductase gene), *fabA* (3-hydroxydecanoyl-ACP dehydratase gene) or *fabZ* (3-hydroxyacyl-ACP dehydratase gene) were not identified but genes encoding proteins from the respective families were present.

### Other Genomic Features

Although flagella were not observed in negatively stained electron microscopy specimens, tumbling motility was sometimes observed in the exponential growth phase (Miroshnichenko et al., 2003). Genome analysis revealed all genes encoding proteins involved in bacterial flagellum formation (Cabys_1463-1503). Pili IV cluster was also found (Cabys_2584-2567), while *Bacteroidetes*-type gliding motility machinery was not.

In total 33 genomic islands were identified in the genome of *C. abyssi* by SWGIS and IslandViewer programs. Distribution of the genomic islands is shown in Supplementary Figure 2. Many genomic islands were located next to tRNA genes and the *rrn* operon. The genetic repertoire was typical for mobile elements with an abundance of plasmid and phage associated genes and multiple genes coding hypothetical proteins. Among proteins with predicted functions there were carbohydrate and lipid metabolism enzymes, metabolic oxidoreductases and hydrolases, several efflux pumps, heavy metal and antimicrobial resistance proteins. The horizontal gene acquisition by *C. abyssi* may provide additional stress response and resistance mechanisms, necessary for adaptation to the instable environment. Genomic islands of *C. abyssi* showed compositional and sequence similarities to many other genomic islands of extremophilic and lithotrophic bacteria in the genera *Dehalococcoides, Thiobacillus, Thermoanaerobacterium, Thermus, Hyphomicrobium, Desulfovibrio*, etc; and also to genomic islands in cyanobacteria such as *Cyanothece, Anabaena, Nostoc* and *Spirosoma*. Strange enough, but some level of compositional similarity was shared with pathogenicity genomic islands of *Salmonella* and *Escherichia*, which was consistent with the hypothesis assuming the origination of antimicrobial genomic islands in human pathogens from marine micro-flora due to the increased level of ocean pollution (Bezuidt et al., 2011).

The *C. abyssi* genome contains multiple copies of an 18-nucleotide repeat with the consensus motif aAACCGTTgaAACGGTTt. Fourteen palindromic positions in this motif (2–8 and 11–17) are highly conserved; the pattern nAACCGTTnnAACGGTTn (further referred to as the palindrome core) was found 38 times as an exact match. Motif occurrences (sites) with up to four mismatches in the core region are also overrepresented in the genome, but positions 1, 9, 10, and 18 are conserved only when no more than one mismatch is allowed in the core (Supplementary Table 7). No significant sequence conservation was observed outside the 18-nucleotide window, therefore these sites are not parts of larger genomic repeats. The sites are distributed rather uniformly along the genome, however, sites with zero mismatches in the core do show weak clustering (Supplementary Figure 3). Zero-mismatch sites are probably functional, because they share common properties such as preference toward intergenic regions, avoidance of gene upstream regions, and strand specificity (for sites with one mismatch, these conclusions are tentative due to insufficient data). Indeed, while 88% of *C. abyssi* genome is covered by predicted CDSs, only 13 of the 38 zero-mismatch sites overlap with coding regions (Supplementary Table 8). All these 13 CDSs are short ORFs with no homologs in the NCBI non-redundant database (Supplementary Table 9), and therefore, most probably have been misannotated. The only CDS containing a one-mismatch site is Cabys_0026, homologous to WP_012842800 (hypothetical protein, *Rhodothermus marinus*). However, the palindromes are unlikely to act as transcription factor binding sites (TFBS), because zero-mismatch sites are rarely found in the intergenic intervals between divergently located genes (start to start), which are typically enriched in regulatory sequences. On the contrary, nine sites have been found between convergently located genes (stop to stop), where no TFBSs are expected (Supplementary Table 10). Notably, zero- and one-mismatch sites, in spite of being almost exact palindromes, seem to be strand-specific, with directionality defined by the two central positions. The two most frequent dinucleotides in positions 9–10, GA and TC (24 and 10 cases out of 43, respectively) are mutually complementary, while other dinucleotides (5 AA, 3 TT, and 1 TA) could all be derived from GA or TC via one substitution. The dinucleotides GA and AA are overrepresented in the first half of the genome, while TC and TT are mainly concentrated in the second half, starting from the predicted origin of replication (Supplementary Table 11 and Supplementary Figure 4). This may indicate motif preference toward the leading or lagging strand.

Such properties as the high copy number, conserved palindromic motif, preference toward intergenic regions, and probable location downstream of genes relate this motif to the repetitive extragenic palindrome (REP) sequences first described in *E. coli* (Stern et al., 1984) and subsequently characterized in many bacteria. The function of these sequences is still controversial but they have been shown to be involved in mRNA stabilization (Newbury et al., 1987; Khemici and Carpousis, 2004), and to act as binding sites for DNA polymerase I (Gilson et al., 1990), DNA gyrase (Espéli and Boccard, 1997) or Integration Host Factor (Engelhorn et al., 1995). However, in *C. abyssi*, conserved intergenic motifs are shorter than typical REP-sequences and are found in lower copy number. Moreover, strand preference has not been described for the REPs, but it is known to be critical for other important genomic motifs like Chi sites involved in homologous recombination (Smith et al., 1981). All these sequences are typically species or genus specific, therefore availability of closely related genomes is essential for further analysis.

### Regulation

Comprehensive analysis of regulatory sequences in *C. abyssi* was complicated by its deep phylogenetic position as the only phylum representative with a sequenced genome. Here we focus only on a few cases, when functional annotations based on sequence homology and genome context were supported by the presence of the corresponding regulatory sequence motifs. These are putative transport systems for nickel, cobalamin, thiamine, and molybdate.

First, two genomic loci (Cabys_1316-1319 and Cabys_2622-2627) share a common upstream sequence motif reminiscent of known NikR binding sites (Supplementary Table 12). The loci contain nickel-responsive regulator *nikR* (Cabys_1316), Ton-B dependent receptors (Cabys_1319 and Cabys_2622) and homologs of *cbi/nikMNOQ* transport systems (Cabys_1317-1318 and Cabys_2625-2627). CbiMNQO and NikMNQO are considered to be the most widespread groups of microbial transporters for cobalt and nickel ions, and their substrate specificity can usually be predicted from the genome context and regulation by the B_12_ riboswitch or NikR repressor, respectively (Rodionov et al., 2006). In *C. abyssi*, the corresponding genes were annotated as *nikMNQO*.

Second, a B_12_ (cobalamin) riboswitch was found to regulate the transport system for cobalamin (vitamin B_12_). As discussed above, *C. abyssi* does not encode genes for *de novo* cobalamin biosynthesis, and probably does not require import of cobalt ions. The predicted cobalamin transport system in the organism is composed of the outer-membrane transport complex (outer membrane TonB-dependent porin BtuB plus the [TonB][ExbB][ExbD] complex) and ABC transporter BtuCDF. A cobalamin riboswitch [AdoCbl RNA motif, Vitreschak et al. (2003)] occurs 292 nucleotides upstream of the candidate operon (Supplementary Figure 5A), which encodes the predicted TonB-dependent porin BtuB and B_12_-binding component BtuF (Cabys_4039-4041). The ATP-binding component BtuD (Cabys_0349) is encoded close to cob(II)alamine reductase (Cabys_0348), cob(I)alamin adenosyltransferase (Cabys_0354), coenzyme B_12_-dependent ribonucleotide reductase complex (Cabys_0352-0353), and ethanolamine utilization cluster (Cabys_0345-0347). The gene of the predicted membrane subunit BtuC (Cabys_1071) is located separately.

Next, a thiamine pyrophosphate riboswitch (TPP, Rodionov et al., 2002) is located 163 nucleotides upstream of the putative TonB-dependent receptor gene (Supplementary Figure 5B); the locus also contains a sodium-solute symporter and a thiamin pyrophosphokinase (Cabys_3975-3977). This TonB-dependent receptor is highly similar to the BT2390 thiamine receptor in *Bacteroides thetaiotaomicron* (Rodionov et al., 2002), and is probably involved in the thiamine transport in *C. abyssi.*

Finally, a molybdenum cofactor riboswitch (Moco RNA motif, Weinberg et al., 2007) was found 1130 nucleotides upstream (Supplementary Figure 5C) of the gene of a predicted molybdate transporter Cabys_2008 (PF16983: Molybdate transporter of MFS superfamily), which lies in the molybdenum cofactor biosynthesis locus (Cabys_2007-2011) coding for proteins MoaA, MoaC, MoeA and MOSC-domain protein [predicted sulfur-carrier domain, present in metal-sulfur cluster biosynthesis proteins, (Anantharaman and Aravind, 2002)].

### Calditrichaeota, a Deep Phylogenetic Lineage

**FIGURE 3:**
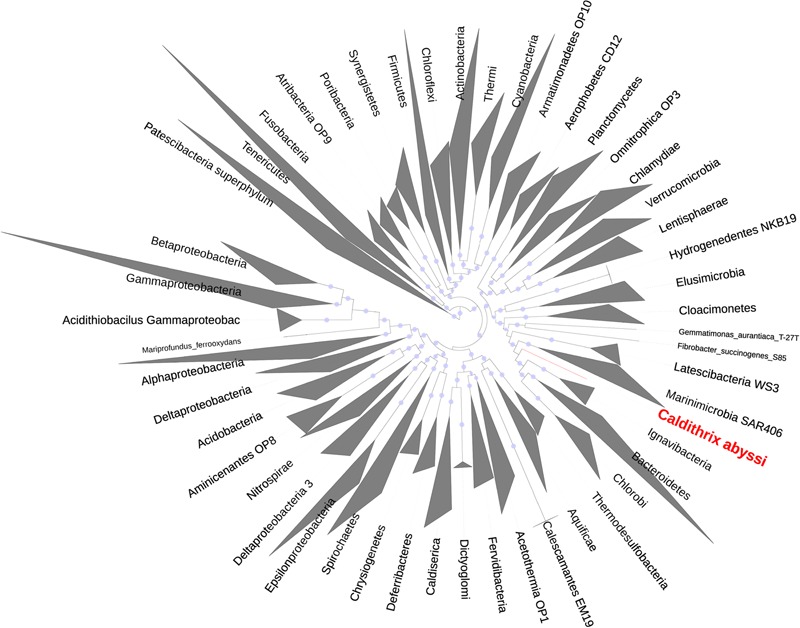
**Maximum likelihood phylogenetic tree based on alignments of 38 marker protein sequences of 2306 bacterial taxa.** *Caldithrix abyssi* LF13, a representative of *Calditrichaeota*, is shown in red. Bar, number of expected amino-acid substitutions per site.

Our phylogenomic analysis with different taxa configurations (**Table 1**) and up to 38 (Supplementary Table 13) and 120 (Supplementary Table 14) marker proteins placed *C. abyssi* in a separate phylum, for which we suggest the name *Calditrichaeota*, within the *Fibrobacteres*–*Chlorobi*–*Bacteroidetes* (FCB) superphylum (Gupta, 2004; **Table 1**). Our results support recent proposal of the addition of the phyla *Marinimicrobia* (SAR406), *Latescibacteria* (WS3), *Cloacimonetes* (WWE1), *Gemmatimonadetes* and the phylum represented by *Caldithrix* to the FCB superphylum (Rinke et al., 2013). In our analysis, *Calditrichaeota* was placed as a sister clade of a group of the phyla *Ignavibacteriae, Chlorobi*, and *Bacteroidetes* (Podosokorskaya et al., 2013) in the majority of the phylogenetic trees (**Figure 3**, Supplementary Figure 6). However, the bootstrap support for the sister-group relationship between *Calditrichaeota* and the three phyla was low (**Figure 3**) and thus the clarification of the exact lineage delineations within the FCB needs to be tested once more genomes become available. The placement of *Calditrichaeota* within the FCB superphylum is further supported by comparative genomic analyses showing that a conserved carboxy-terminal domain of extracellular proteinases (TIGR04183) is found exclusively (but not universally) in members of the FCB superphylum including *Calditrichaeota* (Rinke et al., 2013).

**Table 1:** Phylogenetic inferences and taxa configurations of marker gene trees.

Tree	Domain	Strains	Config	Sister	Superphylum
**a**	BA	2506	all	*Ignavibacteriae, Chlorobi, Bacteroidetes*	FCB
**b**	B	2303	all	*Ignavibacteriae, Chlorobi, Bacteroidetes*	FCB
**c**	BA	2318	gt4kb	*Marinimicrobia*	FCB
**d**	B	2159	gt4kb	*Ignavibacteriae, Chlorobi, Bacteroidetes*	FCB
**e**	BA	2417	gt2kb	*Ignavibacteriae, Chlorobi, Bacteroidetes*	FCB
**f**	B	2238	gt2kb	*Ignavibacteriae, Chlorobi, Bacteroidetes*	FCB
**g**	BA	2252	30g	*Ignavibacteriae, Chlorobi, Bacteroidetes*	FCB
**h**	B	2099	30g	*Ignavibacteriae, Chlorobi, Bacteroidetes*	FCB
**i**	BA	2360	20g	*Marinimicrobia*	FCB
**j**	B	2186	20g	*Ignavibacteriae, Chlorobi, Bacteroidetes*	FCB

*Different strain selections were employed to investigate the position of *Caldithrix abyssi* LF13 in the phylogenetic tree of life. The strain selections were the following: (1) all strains (all), comprising 203 archaeal and 2,303 bacterial genomes; (2) reduced strain sets as based on the alignment length of equal to or greater than 4000 (gt4kb) or 2000 (gt2kb) amino acids; (3) reduced strain set as based on the number of marker genes present, with equal to or greater than 30 (30g) or 20 (20g) marker genes. Abbreviations: tree, phylogenetic tree; B, domains Bacteria; A, domains Archaea, or both (BA); Strains, number of strains used to infer the tree; config, strain selections as described above; sister, sister group of *C. abyssi* LF13 in tree; superphylum, superphylum in which *C. abyssi* LF13 is placed.*

## Discussion

Genome analysis of *C. abyssi* strain LF13 provided a more precise characterization of its metabolic potential and resolved its unique phylogenetic position. Initially *C. abyssi* was described as an organism capable of organoheterotrophic fermentative growth on peptides or nitrate respiration with acetate or lithoheterotrophic growth with molecular hydrogen and nitrate. It was proposed that these capacities allow *C. abyssi* to fit into different food webs in the deep-sea hydrothermal environments. It can use molecular hydrogen of abiotic origin, degrade organic substrates or perform, via nitrate respiration, a complete mineralization of organic matter, oxidizing acetate – the most common partially oxidized product of organic matter degradation.

Our analysis provided the genomic basis of all of the catabolic processes revealed in the initial growth experiments (Miroshnichenko et al., 2003), as well as new insights into the *C. abyssi* metabolism.

The versatility of the *C. abyssi* metabolism is further demonstrated by the presence of gene clusters encoding at least five hydrogenases: three [NiFe]- and two [FeFe]-hydrogenases. The capacity of *C. abyssi* for lithoheterotrophic growth with H_2_ as electron donor, reported by Miroshnichenko et al. (2003), is further supported by the presence in the genome of genes for the uptake [NiFe]-hydrogenase and the bidirectional NAD(P)-dependent [NiFe]-hydrogenase. The reverse process, the disposal of the reducing equivalents produced in the course of fermentation, involves the NAD(P)-dependent bidirectional [NiFe]-hydrogenase and the NADH- and ferredoxin-dependent electron-bifurcating [FeFe]-hydrogenase, with their impacts depending on hydrogen concentration. *C. abyssi* lacks an energy-converting hydrogenase. However, conversion of the energy of the reaction Fd_red_ → H_2_ to the energy of transmembrane potential may occur via operation of the bypass involving the ion-translocating ferredoxin:NAD-oxidoreductase Rnf. As for direct, non-energy-conserving hydrogen production from reduced ferredoxin, which may be pertinent at high H_2_ concentrations, it is probably provided for by those two [NiFe]- and [FeFe]-hydrogenases, the specificities of which we failed to definitely establish because of the lack of biochemically characterized close homologs. The former of these hydrogenases belongs to the group 3c of [NiFe]-hydrogenases. Hydrogenases of this group were previously found only in archaea (Vignais and Billoud, 2007; Peters et al., 2015); however, our blastp searches in the GenBank and IMG databases showed that related hydrogenases also occur in some bacteria. Rather unexpectedly, this *C. abyssi* gene cluster also includes upstream genes of heterodisulfide reductase, a clustering pattern observed in methanogenic archaea, where the two enzymes are involved in the reduction of CoB-S-S-CoM (formed from CH3-S-CoM and HS-CoB in a methane-generating reaction) to HS-CoM and HS-CoB. Such gene clustering pattern also occurs in some bacteria, e.g., *Thermoanaerobaculum aquaticum* and *Candidatus* Acetothermum autotrophicum, where the cluster’s functions are yet unknown.

While the high number of predicted peptidases correlates with fermentative growth of *C. abyssi* on complex proteinaceous substrates, the discovery of 10s of GHs, PLs and CEs, including extracellular ones, did not correlate with the results of previous growth experiments (Miroshnichenko et al., 2003). The GHs, encoded in the genome, belonged to the families of CAZymes (Cantarel et al., 2009) with the following specificities: amylases/pullulanases and alpha-glucosidases, endoglucanases and beta-glucosidases, xyloglucanases, mannosidases, galactosi dases, sucrose phosphorylases, invertases, and some others (Supplementary Table 1). However, our growth experiments that followed these genomic findings and involved various saccharides led to the discovery of only four growth-supporting substrates: starch, cellobiose, xyloglucan and glucomannan. We assume the lack of other activities (e.g., chitinase, cellulase, invertase activities) to be due to faults in gene expression or in enzyme activation or in the transport of substrates/extracellular hydrolysis products into the cells; or, after all, the activities were wrongly predicted.

The genomic analysis of central carbohydrate metabolism of *C. abyssi* revealed all genes of the EM pathway and the synthetic PP pathway, while the ED pathway seems to be inoperative (Supplementary Tables 3–5).

The oxidative TCA cycle is complete in *C. abyssi* (**Figure 1**, Supplementary Table 5). Interestingly, the conversion of 2-oxoglutarate to succinyl-CoA can be accomplished by both 2-oxoglutarate dehydrogenase and 2-oxoglutarate synthase. The former catalyzes irreversible conversion of 2-oxoglutarate to succinyl-CoA and mainly occurs in aerobic bacteria, while the latter is reversible, extremely sensitive to oxygen, and used mainly by anaerobes, albeit with some exceptions (Baughn et al., 2009). The presence of both enzymes might reflect a recent loss by *C. abyssi* of the capacity for aerobic growth.

The *C. abyssi* genome contains all genes of the respiratory electron transfer chain. A NADH dehydrogenase (Complex I) was found, as well as a succinate dehydrogenase/fumarate reductase (Complex II). *C. abyssi*’s complex III (ACIIIcc) locus differs from the previously reported canonical ACIIIcc gene cluster (Refojo et al., 2013), as auxiliary subunits F and G are absent while *c*-type monoheme subunit ActE is duplicated. These cytochromes are expected to transfer electrons to the terminal oxygen reductase, which, however, seems to be inoperative in *C. abyssi* due to rearrangements in its gene cluster (see below).

*Caldithrix abyssi*’s nitrate reduction is determined by the periplasmic dissimilatory nitrate reductase Nap and a homolog of the nitrite reductase 𝜀Hao, recently shown to perform this function in the 𝜀-proteobacterium *Nautilia profundicola* (Simon et al., 2011). The nitrite reductase function of this protein in *C. abyssi* is supported by the lack of the tyrosine protein ligand characteristic of hydroxylamine oxidoreductases of nitrifiers (Simon et al., 2011) and by co-localization of the 𝜀*Hao* gene and the *Nap* gene cluster in *C. abyssi* genome, as it was previously observed in several 𝜀-proteobacteria (Simon and Klotz, 2013) and *Denitrovibrio acetiphilus* DSM 12809 from the phylum *Deferribacteres*. The electron transfer between the quinol pool and periplasmic nitrate reductase NapA in *C. abyssi*, lacking NapB and NapC redox proteins, could be provided via periplasmic tetraheme cytochrome NapM accepting electrons from the Rieske/cytochrome *b* complex, encoded in the Nap cluster (**Figure 1**). Alternatively, electrons could be transferred directly from the quinol-oxidizing NapGH complex to NapA as it was previously proposed for *D. desulfuricans* (Marietou et al., 2005). The electron transfer to the terminal nitrite reductase 𝜀Hao in *C. abyssi* could be provided via the following pathways (**Figure 1**): (i) from the Rieske/cytochrome *b* complex via the NapM periplasmic tetraheme or directly to the octaheme 𝜀Hao; (ii) from ACIIIcc complex via NapM or a chain including plastocyanin and NapM; (iii) via direct interaction of ACIIIcc with the 𝜀Hao. A theoretical possibility of ACIIIcc direct interaction with periplasmic multiheme electron acceptors has already been discussed (Refojo et al., 2012). Electrochemical characteristics and structural features of Rieske proteins (Schneider and Schmidt, 2005; Liu et al., 2014) suggest the possibility of the electron transfer from them to periplasmic multiheme cytochromes *c* (whether NapM or 𝜀Hao). Moreover, *c*-type multihemes have been proposed to serve as electron acceptors for Rieske protein subunits in early evolutionary forms of anaerobic cytochrome *bc* complexes (Dibrova et al., 2013).

So far, there are no evidences, supporting the abilities of the periplasmic nitrate reductase Nap and the nitrite reductase 𝜀Hao to generate transmembrane proton gradient (in other words, to be electrogenic, Simon and Klotz, 2013). However, the generation of *pmf* (proton motive force) in *C. abyssi* could be accomplished by the Rieske/cytochrome *b* or the ACIIIcc complex. The former is well known to be electrogenic (ten Brink and Baymann, 2014) while the latter was proposed by Refojo et al. (2012) to translocate protons in case its FeS-binding ActB subunit faces the cytoplasm and allows electron bifurcation to an external high potential and an internal low potential electron acceptor. In *C. abyssi*, the ActB subunit lacks any signatures of translocation across the cytoplasmic membrane, which indicates its intracellular localization and theoretical possibility of *pmf*-generating activity of ACIIIcc. In this case oxidized ferredoxin could act as an internal electron acceptor for the ACIIIcc. Anyway, regardless of the ability of the Rieske/cytochrome *b* or ACIIIcc complexes to be electrogenic, the *pmf* will be generated due to the action of NADH dehydrogenase (complex I) during growth with acetate and nitrate. As to the lithotrophic growth, the enzymatic complexes oxidizing hydrogen and reducing nitrate and nitrite are as a whole electrogenic in *C. abyssi* (again, regardless of the possible electrogenicity of ACIIIcc). The arguments are as follows. Like other [Ni,Fe]-hydrogeases of group 1 (Vignais and Billoud, 2007), the uptake hydrogenase of *C. abyssi* should oxidize hydrogen and produce protons in the periplasm and transfer electrons to quinones via its cytochrome *b* subunit. Analysis of the primary and secondary structure of this subunit provides us grounds to predict that the accompanying protons are received by quinones from the cytoplasm (to be eventually carried out to the periplasm), as it was inferred for the uptake group 1 [Ni,Fe]-hydrogenase of *Wolinella succinogenes* from targeted mutagenesis studies (Gross et al., 2004). The total balance of protons in the systems that include this hydrogenase and Nap or 𝜀Hao makes these systems electrogenic. As for the bidirectional group 3 [Ni,Fe]-hydrogenase, the electrogenic nature of the nitrate- and nitrite-reducing systems, that include it, should be provided for by complex I, which oxidizes NADH, produced by this hydrogenase, and pumps out protons at the expense of this reaction.

The initial growth experiments (Miroshnichenko et al., 2003) had shown that nitrate was the only electron acceptor that supported growth of *C. abyssi* on non-fermentable substrates. The current genomic analysis suggested possible capacities for the respiratory growth with sulfur/polysulfide/thiosulfate, tetrathionate, and oxygen as the electron acceptors; however, the subsequent growth experiments failed to support these predictions. Elemental sulfur and thiosulfate could not serve as electron acceptors for growth with hydrogen or acetate, although, when peptone was used as the substrate, the first two compounds were reduced to sulfide and slightly stimulated fermentative growth (Supplementary Table 2). The lack of sulfur and thiosulfate respiration might be explained by a non-operational state of molybdopterin polysulfide/thiosulfate reductase, probably due to the lack of the TorD protein, which plays a crucial role in the maturation of the subunit A and whose absence leads to significant (Ilbert et al., 2003) or complete (Shaw et al., 1999) loss of subunit A activity. The stimulation of fermentative growth may be a result of action of cytoplasmic sulfide dehydrogenase (Ma and Adams, 1994; Bridger et al., 2011; Schut et al., 2013) or bidirectional NAD(P)-dependent [NiFe]-hydrogenase, also capable of sulfur reduction (Ma et al., 1993; Schut et al., 2013). This non-energy-conserving reduction of the external electron acceptor may stimulate the growth due to disposal of excess of reducing equivalents generated during fermentation (Ma et al., 1993).

*Caldithrix abyssi* possesses a complete set of genes encoding the terminal, heme *a*- and copper-containing oxygen reductase (respiratory complex IV). Our analysis revealed that, among *Bacteria*, chromosomal cassettes containing heme-copper cytochrome *c* oxidase genes (*coxI-IV, cox15*, farnesyltransferase gene) are present only in organisms capable of aerobic respiration (Supplementary Table 6). Almost all of these aerobic, microaerophilic and facultatively anaerobic bacteria harboring the discussed chromosomal cassettes exhibit a conserved gene order (Supplementary Figure 1). *C. abyssi* is one of three exceptions among the 109 strains retrieved by our screening procedure, and the only of them that is incapable of aerobic growth. In contrast to the others, its cytochrome *c* oxidase gene cluster contains only one copy of the *coxIII* gene, and the genes of the heme-processing enzymes heme *a* synthase and farnesyltransferase (*cox15* and *ctaB*) are located separately from each other and, in the case of *ctaB*, well upstream of *coxI*, encoding the catalytic heme-containing subunit. We suppose that such differences in the order of core genes responsible for the maturation of cytochrome *c* oxidase lead to a wrong assembly of the complex IV in *C. abyssi* and, as a consequence, to the inability of this organism to respire oxygen. Considering the homologous oxygen reductase from a strict anaerobe *Desulfovibrio vulgaris* str. Hildenborough, involved exclusively in oxygen detoxification (Lamrabet et al., 2011), we cannot exclude oxygen detoxifying function of the complex IV in *C. abyssi*. However, the observed recombination of the heme-processing genes could prevent any kind of oxygen-reducing activity of the enzyme complex. The resistance of *C. abyssi* to microaerobic conditions may be due to catalase/peroxidase.

Based on the genomic analysis, *C. abyssi* is likely to be prototrophic for all amino acids and nucleotides. However, several pathways (lysine, arginine and serine biosynthesis) need further investigation, as they may involve non-orthologous gene displacements or are after all incomplete. *C. abyssi* is most probably auxotrophic for some vitamins and cofactors, such as biotin, thiamine, and cobalamin. In the case of thiamine and cobalamin, candidate transporters were identified. Likely prototrophy for fatty acids is inferred from the presence of the fatty-acids biosynthesis gene cluster conserved in many bacteria. However, some genes from the pathway were not identified.

In general, analysis of regulatory interactions is a powerful method for bacterial genome annotation. Identification of riboswitches and TFBS allowed us to annotate thiamine, cobalamin, nickel, and molybdate transporters. However, the power of this analysis is presently limited by the fact that *C. abyssi* is currently the only representative of the proposed phylum *Calditrichaeota* whose completely sequenced genome is available.

One more interesting feature of the *C. abyssi* genome that needs to be further investigated by comparative analysis with other phylum representatives is a highly conserved 18-nucleotide motif. As discussed above, some of its properties make it related to the REP-sequences or Chi sites, though annotation of such motifs is complicated because their sequences are conserved only in closely related bacteria.

## Conclusion

To conclude, *C. abyssi* is a metabolically versatile representative of a deep, phylum-level bacterial lineage. The phylum level depth of branching of this lineage was already proposed in the very first paper (Miroshnichenko et al., 2003) and was confirmed in recent phylogenomic studies such as that by Rinke et al. (2013). Here we claim a novel phylum based on comprehensive phylogeny involving 38 proteins. The detailed metabolic reconstruction of its first representative revealed a much more diverse functional repertoire compared to the original description, made more than a decade ago. Moreover, the originally provided genomic G+C content value turned out to be too imprecise (Meier-Kolthoff et al., 2014). Taking into account the recent authoritative suggestion to include the rank of phylum in the International Code of Nomenclature of Prokaryotes (Oren et al., 2015), we present a section “Description of *Calditrichaeota* phyl. nov.,” along with descriptions of fam. nov., ord. nov. and class. nov. based on the emended description of the genus.

### Description of *Calditrichaeota* phyl. nov.

*Calditrichaeota* (Cal.di.tri.chae.o‘ta N.L. pl. n. the phylum of the genus *Caldithrix*). The type class is *Calditrichae*. The phylum- level lineage was already mentioned in the original description of the genus *Caldithrix* (Miroshnichenko et al., 2003) and was based on 16S rRNA gene-based phylogenetic analysis of *C. abyssi*, one environmental clone and representatives of other phyla. Here, the phylum *Calditrichaeota* is introduced based on the phylogenetic analysis of 38 universally conserved single-copy proteins present in *Bacteria* and *Archaea* (Rinke et al., 2013). Currently, the phylum is represented by the class *Calditrichae* class. nov., the order *Calditrichales* class. nov., the family *Calditrichaceae* fam. nov., and the genus *Caldithrix*. The description is based on Miroshnichenko et al. (2010) and the emendations indicated below.

### Description of *Calditrichae* class. nov.

*Calditrichae* (Cal.di’tri.chae N.L. fem. pl. n. *Caldithrix*, type genus of the type order of the class; *Calditrichae*, the class of the genus *Caldithrix*). The description is the same as for the type order *Calditrichales* ord. nov.

### Description of *Calditrichales* ord. nov.

*Calditrichales* (Cal.di.tri’cha.les N.L. masc. n. *Caldithrix* type genus of the order; N.L. fem. pl. n.*Calditrichales* order of the genus *Caldithrix.* The description is the same as for the family *Calditrichaceae* fam. nov.

### Description of *Calditrichaceae* fam. nov.

*Calditrichaceae* (Cal.di.tri’cha.ce.ae. N.L. masc. n. *Caldithrix*, type genus of the family; -aceae ending to denote a family; N.L. fem. pl. n. *Calditrichaceae* family of the genus *Caldithrix*).

### Emended Description of the Genus *Caldithrix* Miroshnichenko et al. (2010)

The description is based on that provided in Miroshnichenko et al. (2010) with the same emendations as for *C. abyssi* (see below).

### Emended Description of the Species *C. abyssi* Miroshnichenko et al. (2003)

In addition to the properties given in the original description of the species (Miroshnichenko et al., 2003), *C. abyssi* is able to grow with starch, cellobiose, glucomannan, and xyloglucan as the substrates. S° or thiosulfate stimulate fermentative growth on peptides and are reduced to sulfide. The *in silico* calculated genomic DNA G+C content of the type strain is 45.1 mol%.

## Author Contributions

OK and MM contributed to microorganism cultivation and characterization. NC, CD, TR, NI, MG, and NK contributed to genome sequencing and assembly. IK, SG, OS, AL, CR, SS, OR, TW, and MSG contributed to genome analysis. IK, H-PK, TW, MSG, and EB-O designed the work. IK, OS, SG, AL, MM, TW, MG, MSG, and EB-O wrote the manuscript.

## Conflict of Interest Statement

The authors declare that the research was conducted in the absence of any commercial or financial relationships that could be construed as a potential conflict of interest.

## Footnotes

1 http://phylosift.wordpress.com/
