# Supplementary material for: Genomic Analysis of Caldithrix abyssi, the Thermophilic Anaerobic Bacterium of the Novel Bacterial Phylum Calditrichaeota
Source: Front Microbiol. 2017 Feb 20;8:195. doi: 10.3389/fmicb.2017.00195 (PMC5317091; doi:10.3389/fmicb.2017.00195)
Supplement: Supplementary file 1 [file Presentation_1.pdf]

Supplementary Table 1. Glycoside hydrolases (GHs), encoded in the genome of *C. abyssi*.

#	Gene id, Caby	protein family	Predicted protein function	Length, aa	Organism with closest homolog	% of identity/ % of coverage	Taxonomy of other closest hits
1.	23	GH13	pullulanase or cyclomaltodextrinase, not amylase	616	uncultured soil bacterium	58/94	Mros_0762/SAR406/Bacteroidetes /Proteo/Firmicutes
2.	35	GH13	a-amylase	441	Caldithrix abyssi 0170	43/73	Proteo/Bacteroidetes/->Thermotogae/Archaea
3.	37	GH13	Malto-oligosyltrehalose trehalohydrolase or 1,4-alpha-glucan branching enzyme	964	Ignavibacterium album	51/99	Ignavibacteria/Bacteroidetes
4.	170	GH13	Nd	464	Odoribacter sp. CAG:788 (Bacteroidetes)	41/88	Bacteroidetes/Proteo
5.	1003	COG2152/DUF377	Nd	416	Fibrisoma limi (Bacteroidetes)	44/99	Proteo/Bacteroidetes
6.	1004	COG2152/DUF377	Nd	306	Caldithrix abyssi 2432	48/97	Proteo/Firmicutes
7.	1006	GH2	b-mannosidase	813	Dictyoglomus turgidum	42/74	Actinobacteria/Firmicutes
8.	1350	GH1	b-glucosidase	426	Melioribacter roseus P3M-2 (Mros_0638)	50/98	Proteo/Thermotogae/Firmicutes/Thermotogae & Euryarchaeota
9.	1626	GH13	pullulanase or cyclomaltodextrinase	600	Melioribacter roseus P3M-2 (Mros_0762)	52/96	Bacteroidetes/Firmicutes and many other
10.	1880	GH13	sucrose phosphorylase	627	delta proteobacterium NaphS2	59/98	many diff phyla
11.	1956	GH2	Exo-beta-D-glucosaminidase	863	SAR406 cluster bacterium (JGI)	63/100	Rhodothermus/Ignavibacteria/other Caldithrix
12.	1959	GH9	endoglucanase	672	Ignavibacterium album JCM 16511	44/100	Meliori (MROS_0757)/Firmicutes
13.	1960	GH18	Nd	454	SAR406 cluster bacterium (JGI)	40/98	uncultured, candidatus, archaea, insecta
14.	1963	DUF1083	GH putative		SAR406 cluster bacterium (JGI)	51/74	Ignavibacteria/SAR406/Bacteroide

							tes
15.	1968	GH3	beta-glucanase (Swiss)	749	Xenococcus sp. PCC 7305 (Cyanobacteria)	59/96	Proteo/Bacteroidetes
16.	1971	GH5	Nd	346	Anaerolinea thermophila	61/87	Thermotogae/Dictyoglomi
17.	2099	GH13	a-amylase (Swiss)	779	Daphnia pulex (Eukarya)	57/85	few Daphnia/1 Bacteroidetes/Firmicutes
18.	2101	GH3	Nd	836	Ignavibacterium album JCM 16511	45/97	Bacteroidetes
19.	2116	GH16	Nd	609	Ignavibacterium album JCM 16511	41/90	Proteo/Bacteroidetes
20.	2338	GH20	Nd	561	Anaerophaga thermohalophila	60/96	Bacteroidetes
21.	2432	COG2152/DUF 377	Nd	322	Deferribacter desulfuricans	63/92	Firmicutes/Bacteroidetes->Archaea
22.	2448	DUF1083	Nd		Caldithrix abyssi 1963	41/99	Ignavibacteria/SAR406/Bacteroidetes
23.	2449	GH5	Nd	633	SAR406 cluster bacterium (JGI)	51/99	majority - Bacteroidetes
24.	2569	GH94	Nd	806	Pseudanabaena sp. PCC 7367 (Cyanobacteria)	53/96	Cyanobacteria/Firmicutes/Ignavibacteria->Thermotogae
25.	2706	GH2	b-galactosidase or Beta-glucuronidase	838	Ignavibacterium album JCM 16511	20/89	Bacteroidetes
26.	2809		Nd	932	Clostridium clariflavum	31/46	
27.	2922	GH18	Nd	825	uncultured bacterium 1250012-L08	37/100	uncultured, Bacteroidetes
28.	3078	GH13	isoamylase	706	delta proteobacterium MLMS-1	55/96	Proteo/Cyanobacteria and many other
29.	4192	GH13	a-glucosidase	1144	Roseiflexus castenholzii	56/83	many different including Mros_1936
30.	4194	GH13	Nd	874	Haloferax mediterranei	27/71	Haloarchaea

Nd – not determined

Supplementary Table 2. Electron donors and acceptors supporting the growth of *C. abyssi*.

Utilization of	Initial description (Miroshnichenko et al., 2003)	Current growth experiments	Genomic prediction
	as electron donors		
Beef, soy bean, peptone, yeast	+	Nd	+
Starch	-	+	+
Maltose	-	-	+?
Pullulan	Nd	-	+
Dextran	Nd	-	+
Dextrin	-	+/-	+
Cellulose	Nd	-	+
Cellobiose	-	+	+
Trehalose	Nd	-	+/-
Lactose	Nd	-	+
Pectin	Nd	-	+/-
Sucrose	-	-	+
Chitin	Nd	-	+
Chitosan	Nd	-	+
Agarose	Nd	-	Nd
Mannobiose/triose	Nd	Nd	+
Alginate	Nd	-	---/+
Glucomannan	Nd	+	+
Xyloglucan	Nd	+	+
	as electron acceptors		
**Nitrate**	+	+	+
Sulfate	-	_	-
S°	-	+*	-/+
Thiosulfate	-	+*	-/+
Nitrite	-	-	+
Arsenate	Nd	Nd	-/+
Tetrathionate	Nd	Nd	+
O_2	Obligate anaerobe	Obligate anaerobe	+/-

*Peptone was used as a substrate. Slight growth stimulation in comparison with control experiments (peptone without S° or thiosulfate) was detected. The growth was not detected on acetate or H_2 with S° or thiosulfate.

Supplementary Table 3. Identified enzymes of the Embden-Meyerhof and gluconeogenesis pathways

**Enzyme**	**Short name**	**EC number**	**Gene ID**	**COG**
<u>ATP hexokinase</u>	<u>HK</u>	2.7.1.2	Caby_1958	COG1940
			Caby_2666	COG1940
			Caby_3485	COG1940
glucose-6-phosphate isomerase	PGI	5.3.1.9	Caby_0862	COG0166
bifunctional mannose-6-phosphate isomerise/glucose-6-phosphate isomerase	PMI/PGI	5.3.1.8/5.3.1.9	Caby_3891	COG0449
ATP-dependent 6-phosphofructokinase	PFK	2.7.1.90	Caby_1204	COG0205
			Caby_1301	COG0205
fructose-1,6-bisphosphatase	*FBP*	3.1.3.11	Caby_2424	COG0158
Fructose bisphosphate aldolase class 1	FBA	4.1.2.13	Caby_1795	COG1830
FBAs class 2	FBA	4.1.2.13	Caby_2273	COG0191
NAD+ Glyceraldehyde-3-phosphate dehydrogenase	GAPDH	1.2.1.12	Caby_2743	COG0057
phosphoglycerate kinase	PGK	2.7.2.3	Caby_2742	COG0126
phosphoglycerate mutase	GPM	5.4.2.12	Caby_0791	COG0696
enolase	ENO	4.2.1.11	Caby_2868	COG0148
<u>pyruvate kinase</u>	<u>PK</u>	2.7.1.40	Caby_0156	COG0469
pyruvate, phosphate dikinase;<br>archaeal type	PPDK	2.7.9.1	Caby_1403	COG0574
			Caby_3890	Nd
phosphoenolpyruvate synthetase;	*PEPS*	2.7.9.2	Caby_1553	COG0574
			Caby_1123	COG0574
			Caby_1159	COG0574

EM only: underlined. Gluconeogenesys only: italic.

Nd – not determined

Supplementary Table 4. Identified enzymes of the pentose-phosphate pathway and of subsequent pentose conversions

Enzyme	Short name	EC number	Gene ID	COG
		Pentose-phosphate pathway		
Transketolase	TKT	2.2.1.1	Caby_1560	COG0021
Transaldolase	TAL	2.2.1.2	Caby_1563	COG0176
			Caby_1564	COG0176
Ribulose-phosphate 3-epimerase	RPE	5.1.3.1	Caby_2787	COG0036
Ribose-5-phosphate isomerase	RpiB	5.3.1.6	Caby_2247	COG0698
			Caby_4204	COG0698
		Subsequent pentose conversions		
Ribokinase, putative	RBSK	2.7.1.15	Caby_1685	COG0524
Ribose-phosphate pyrophosphokinase	RPPK	2.7.6.1	Caby_4008	COG0462
Phosphopentomutase	DeoB	5.4.2.7	Caby_1186	COG1015

Supplementary Table 5. Identified enzymes of the oxidative tricarboxylic acid (TCA) cycle and associated reactions

Enzyme	Short name	EC number	Gene ID	COG
		Associated reactions		
Pyruvate dehydrogenase E1 component		1.2.4.1	Caby_3801	COG2609
Pyruvate dehydrogenase E2 component		2.3.1.12	Caby_3800	COG0508
Pyruvate dehydrogenase E3 component		1.8.1.4	Caby_3799	COG1249
Pyruvate ferredoxin oxydoreductase	POR	1.2.7.1	Caby_0262	COG0674+COG1013+COG1014
Malic enzyme (NADP+ dependent)	MAE	1.1.1.40	Caby_2313	COG0280+COG0281
PEP carboxylase (ATP)	PCK	4.1.1.49	Caby_0267	COG1866
		Oxidative TCA		
Citrate synthase	CISY	2.3.3.1	Caby_0022	COG0372
Aconitase	ACO	4.2.1.3	Caby_0082	COG1048
Isocitrate dehydrogenase	IDH	1.1.1.41	Caby_3783	COG0473
		1.1.1.42	Caby_1777	COG2838
2-oxoglutarate dehydrogenase		1.2.4.2	Cab_1911	COG0567

Dihydrolipoyllysine-residue succinyltransferase (component of 2-oxoglutarate dehydrogenase complex)		2.3.1.61	Caby_1912	COG0508
2-oxoglutarate synthase	KOR	1.2.7.3	Caby_1703	COG0674+COG1014
			Caby_1704	COG1013
			Caby_3126	COG0674
			Caby_3127	COG1013
Succinyl-CoA ligase, subunit A	SUC	6.2.1.5	Caby_3467	COG0074
subunit B			Caby_3468	COG0045
Succinate dehydrogenase, subunit A	SDH	1.3.5.1	Caby_4074	COG1053
subunit B			Caby_4075	COG0479
subunit C			Caby_4073	
Fumarate hydratase, subunit A	FUM	4.2.1.2	Caby_0173	COG1951
subunit B			Caby_0172	COG1838
subunit C			Caby_0171	COG0114
Malate dehydrogenase	MDH	1.1.1.37	Caby_3850	COG0039

Supplementary Table 6. Results of the search for chromosomal cassettes containing Cox operon in bacterial genomes from IMG database (on December 27, 2015).

#	Bacterial strain (genome name) in IMG database	Chromosomal cassette type[1]	Analysis type[2]	Metabolic type	Reference[3]	Comments
1	Adhaeribacter aquaticus MBRG1.5, DSM 16391	A	COG terms	Aerobe	IMG Project Info	
2	Algoriphagus alkaliphilus DSM 22703	A	COG terms	Aerobe	IJSEM	
3	Algoriphagus boritolerans DSM 17298	A	COG terms	Aerobe	IJSEM	
4	Algoriphagus faecimaris DSM 23095	A	COG terms	Aerobe	IJSEM	
5	Algoriphagus hitonicola DSM 19315	A	COG terms	Aerobe	IJSEM	
6	Algoriphagus mannitolivorans DSM 15301	A	COG terms	Aerobe	IMG Project Info	
7	Algoriphagus marincola DSM 16067	A	COG terms	Aerobe	IMG Project Info	
8	Algoriphagus ornithinivorans DSM 15282	A	COG terms	Aerobe	Yi et al., 2004. IJSEM 54: 157-162	
9	Algoriphagus sp. PR1	A	COG terms	Aerobe	IJSEM	
10	Algoriphagus terrigena DSM 22685	A	COG terms	Aerobe	IJSEM	
11	Algoriphagus vanfongensis DSM 17529	A	COG terms	Aerobe	IMG Project Info	
12	Algoriphagus zhangzhouensis DSM 25035	A	COG terms	Aerobe	IJSEM	
13	Anditalea andensis LY1	A	COG terms	Aerobe	IJSEM	
14	Belliella baltica BA134, DSM 15883	A	COG terms	Aerobe	IJSEM	
**15**	**Caldithrix abyssi LF13, DSM 13497**	**D**	**COG terms**	**Anaerobe**	**IJSEM**	
16	Cecembia lonarensis LW9	A	COG terms	Aerobe	IMG Project Info	
17	Cesiribacter andamanensis AMV16	A	COG terms	Aerobe	IJSEM	
18	Chryseolinea serpens DSM 24574	A	COG terms	Aerobe	IJSEM	
19	Cytophaga aurantiaca DSM 3654	A	COG terms	Aerobe	Winogradsky, 1929. Annales de l'Institut Pasteur (Paris) 43: 549-633.	
20	Cytophaga hutchinsonii ATCC 33406	A	COG terms	Aerobe	IMG Project Info	
21	Cytophaga hutchinsonii DSM 1761	A	COG terms	Aerobe	Winogradsky, 1929. Annales de l'Institut Pasteur (Paris) 43: 549-633.	
22	Dyadobacter beijingensis DSM 21582	A	COG terms	Aerobe	IJSEM	
23	Dyadobacter crusticola DSM 16708	A	COG terms	Aerobe	IJSEM	
24	Dyadobacter fermentans	A	*cox15*query	Aerobe	BioPrpoject info	CoxIV in the cassette assigned manually

25	Dyadobacter ginsengisoli DSM 21015	A	COG terms	Aerobe	IMG Project Info	
26	Dyadobacter koreensis DSM 19938	A	COG terms	Aerobe	IJSEM	
27	Dyadobacter soli DSM 25329	A	COG terms	Aerobe	IJSEM	
28	Dyadobacter sp. Leaf189	A	COG terms	Aerobe	BioSample info	aerobiosis assumed from culture medium description
29	Dyadobacter sp. SG02	A	COG terms	Aerobe	IMG Project info	
30	Dyadobacter tibetensis Y620-1	A	COG terms	Aerobe	IJSEM	
31	Echinicola vietnamensis KMM 6221, DSM 17526	A	COG terms	Aerobe	IJSEM	
32	Emticicia oligotrophica	A	*cox15*query	Aerobe	BioPrpoject info	CoxIV in the cassette assigned manually
33	Fibrella aestuarina	A	COG terms	Facultative anaerobe	IJSEM	
34	Fibrisoma limi BUZ 3	A	COG terms	Facultative anaerobe	IJSEM	
35	Flexibacter elegans DSM 3317	A	COG terms	Aerobe	IMG Project Info	
36	Fulvivirga imtechensis AK7	A	COG terms	Aerobe	IMG Project Info	
37	Halomonas anticariensis FP35	B	COG terms	Aerobe	IMG Project info	
38	Hongiella marincola HL-49	A	COG terms	Aerobe	IJSEM	
39	Hymenobacter arizonensis DSM 17870	A	COG terms	Aerobe	Reddy & Garcia-Pichel, 2013. Ant van Leeuwenhoek 103: 321-330	aerobiosis assumed from culture medium description
40	Hymenobacter gelipurpurascens AT01-02	A	COG terms	Aerobe	Buczolits et al., 2006. IJSEM 56:2071-2078.	
41	Hymenobacter sp. APR13	A	COG terms	Aerobe	IMG Project Info	
42	Hymenobacter sp. AT01-02	A	COG terms	Aerobe	IMG Project Info	
43	Hymenobacter terrenus sp. MIMtkLc17	A	COG terms	Aerobe	IJSEM	
44	Hymenobacter swuensis DY53	A	COG terms	Aerobe	Lee et al., 2014. Curr Microbiol 68: 305–310	aerobiosis assumed from culture medium description
45	Indibacter alkaliphilus LW1 (Draft1)	A	COG terms	Aerobe	IMG Project Info	
46	Leadbetterella byssophila 4M15, DSM 17132	A	COG terms	Aerobe	IJSEM	
47	Mariniradius saccharolyticus AK6	A	COG terms	Aerobe	IJSEM	
48	Marivirga tractuosa H-43, DSM 4126	A	COG terms	Aerobe	IMG Project Info	

49	Meiothermus chliarophilus ALT-8, DSM 9957	C	COG terms	Aerobe	IJSEM	
50	Microscilla marina	A	*cox15*query	Aerobe	BioPrpoject info	no CoxIV in the cassette
51	Microscilla marina ATCC 23134	A	COG terms	Aerobe	Bergey's Manual of Systematic Bacteriology: 2nd edition. Volume 4. 2010	
52	Mucilaginibacter frigoritolerans ATCC BAA-1854	A	COG terms	Aerobe	IJSEM	
53	Mucilaginibacter paludis TPT56, DSM 18603	A	COG terms	Facultative anaerobe	IMG Project Info	
54	Mucilaginibacter polytrichastri DSM 26907	A	COG terms	Aerobe	IJSEM	
55	Mucilaginibacter sp. OK098	A	COG terms	Aerobe (?)	No data available, presumably aerobic as isolated from aspen root microbiome	
56	Nitritalea halalkaliphila LW7	A	COG terms	Aerobe	IMG Project Info	
57	Olivibacter sitiensis DSM 17696	A	COG terms	Aerobe	IMG Project Info	
58	Pedobacter africanus DSM 12126	A	COG terms	Aerobe	IJSEM	
59	Pedobacter arcticus A12	A	COG terms	Facultative anaerobe	IMG Project Info	
60	Pedobacter borealis DSM 19626	A	COG terms	Aerobe	IJSEM	
61	Pedobacter caeni DSM 16990	A	COG terms	Aerobe	IJSEM	
62	Pedobacter insulae DSM 18684	A	COG terms	Aerobe	IJSEM	
63	Pedobacter kyungheensis KACC 16221	A	COG terms	Aerobe	Yang et al., 2012. J Gen Appl Microbiol 58: 309-316	
64	Pedobacter metabolipauper DSM 19035	A	COG terms	Microaerophile	IJSEM	
65	Pedobacter nutrimenti DSM 27372	A	COG terms	Aerobe	IJSEM	
66	Pedobacter oryzae DSM 19973	A	COG terms	Aerobe	IMG Project Info	
67	Pedobacter piscium DSM 11725	A	COG terms	Aerobe	IJSEM	
68	Pedobacter rhizosphaerae DSM 18610	A	COG terms	Aerobe	IJSEM	
69	Pedobacter saltans Steyn 113, DSM 12145	A	COG terms	Aerobe	IMG Project Info	
70	Pedobacter soli DSM 18609	A	COG terms	Aerobe	IJSEM	
71	Pedobacter sp. BAL39	A	COG terms	Aerobe	BioProject info	

72	Pedobacter sp. Leaf176	A	COG terms	Aerobe	BioSample info	aerobiosis assumed from culture medium description
73	Pedobacter sp. Leaf194	A	COG terms	Aerobe	BioSample info	aerobiosis assumed from culture medium description
74	Pedobacter sp. Leaf41	A	COG terms	Aerobe	BioSample info	aerobiosis assumed from culture medium description
75	Pedobacter sp. OK626	A	COG terms	Aerobe (?)	No data available, presumably aerobic as related to aspen root microbiome	
76	Pedobacter sp. R20-19	A	COG terms	Aerobe (?)	No data available, presumably aerobic as isolated from a freshwater creek	
77	Pedobacter sp. V48	A	COG terms	Aerobe	Bitzer et al., 2014. Genome Announc 2(1): e00094-14	
78	Pedobacter suwonensis DSM 18130	A	COG terms	Aerobe	IJSEM	
79	Pontibacter actiniarum KMM 6156, DSM 19842	A	COG terms	Aerobe	IMG Project Info	
80	Pontibacter akesuensis DSM 18820	A	COG terms	Aerobe	IJSEM	
81	Pontibacter kolensis 206081	A	COG terms	Aerobe	IMG Project Info	
82	Pontibacter korlensis X14-1T	A	COG terms	Aerobe	IJSEM	
83	Pontibacter roseus DSM 17521	A	COG terms	Aerobe	IMG Project Info	
84	Pseudosphingobacterium domesticum DSM 18733	A	COG terms	Aerobe	IMG Project Info	
85	Rhodonellum psychrophilum DSM 17998	A	COG terms	Aerobe	IMG Project Info	
86	Rudanella lutea DSM 19387	A	COG terms	Aerobe	IJSEM	
87	Runella limosa	A	*cox15*query	Aerobe	BioPrpoject info	CoxIV in the cassette assigned manually
88	Runella slithyformis LSU4, DSM 19594	A	COG terms	Aerobe	IJSEM	
89	Solitalea canadensis USAM 9D, DSM 3403	A	COG terms	Facultative anaerobe	IJSEM	
90	Sphingobacteriaceae bacterium DW12	A	COG terms	Aerobe (?)	No data available, presumably aerobic as isolated from aerobic soil	
91	Sphingobacterium antarcticum ATCC 51969	A	COG terms	Aerobe	IJSB	
92	Sphingobacterium mizutaii DSM 11724	A	COG terms	Aerobe	IJSB	

93	Sphingobacterium multivorum DSM 11691	A	COG terms	Aerobe	IJSEM	
94	Sphingobacterium siyangensis CGMCC 1.6855	A	COG terms	Aerobe	IJSEM	
95	Sphingobacterium sp. 21	A	COG terms	Aerobe (?)	Data available on the closest characterized relative Olivibacter jilunii (99% 16S rRNA gene seq. ID at 98% coverage in SILVA database)	
96	Sphingobacterium sp. ACCC 05744	A	COG terms	Aerobe	Teng et al., 2015. PLoS One 10(4): e0122254	
97	Sphingobacterium sp. Ag1	A	COG terms	Aerobe	BioSample info on SAMN03487931	aerobiosis assumed from culture medium description
98	Sphingobacterium sp. H1ai	A	COG terms	Aerobe (?)	No data available, presumably aerobic as isolated from Carnivorous plant phytotelma	
99	Sphingobacterium sp. ML3W	A	COG terms	Aerobe	Smith et al., 2015. Genome Announc 3(1): e01477-14.	
100	Sphingobacterium spiritivorum ATCC 33861	A	COG terms	Aerobe	IMG Project Info	
101	Sphingobacterium thalpophilum DSM 11723	A	COG terms	Aerobe	IMG Project Info	
102	Sphingobacterium wenxiniae DSM 22789	A	COG terms	Aerobe	IJSEM	
103	Spirosoma endophyticum DSM 26130	A	COG terms	Aerobe	IJSEM	
104	Spirosoma linguale DSM 74	A	COG terms	Aerobe	IJSEM	
105	Spirosoma luteum DSM 19990	A	COG terms	Aerobe	IJSEM	
106	Spirosoma panaciterrae	A	*cox15*query	Aerobe	BioPrpoject info	CoxIV in the cassette assigned manually
107	Spirosoma radiotolerans DG5A	A	COG terms	Aerobe	Lee et al., 2014 Curr Microbiol 69: 286–291	
108	Spirosoma spitsbergense DSM 19989	A	COG terms	Aerobe	IJSEM	
109	Sporocytophaga myxococcoides DSM 11118	A	COG terms	Aerobe	DSMZ strain datasheet	aerobiosis assumed from culture medium description

[1] Refer to Supplementary Figure 1 for the description of the chromosomal cassette type (A – typical aerobic, B - Halomonas anticariensis, C – Caldithrix abyssi, D – Meiothermus chliarophilus).

[2] Refer to Materials and Methods section for detailed description of analytical procedures.

[3] IJSEM/IJSB refers to the data retreived from valid description of the taxon name in IJSEM and IJSB journals, we do not provide bibliography for these cases; IMG Project info - data retreived from IMG Project information page for the genome of the strain; BioProject - data retreived from NCBI BioProject database for the BioProject number, indicated in IMG Project information page for the genome of the strain; BioSample - data retreived

from NCBI BioSample database for the BioSample number, indicated in IMG Project information page for the genome of the strain; for other cases special references are provided.

Aerobe(?) – altogether 5 of 109 cases - means that exact physiological information is unavailable, however the sources of isolation of these microorganisms and their taxonomy indicate high probability of aerobic growth.

Supplementary Table 7. Sites with 0 to 4 mismatches in the core region of the palindrome (nAACCGTTnnAACGGTTn).

For the calculation of expected numbers of motifs, all bases were assumed to have equal frequencies. This simplification is not expected to introduce significant biases in the estimates given G+C-content of 45,1%, and a high level of motif overrepresentation.

Expected numbers of motifs are calculated by the formula: $n = N \times \frac{\binom{m}{14}3^m}{4^{14}}$, where N is the genome length (4,978,541 base pairs) and m is the number of mismatches in motif nAACCGTTnnAACGGTTn (positions 1,9,10 and 18 are not constrained).

	0 mismatches	1 mismatch	2 mismatches	3 mismatches	4 mismatches
LOGO	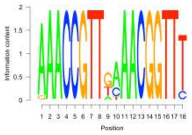	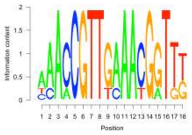	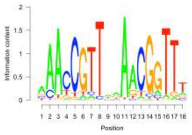	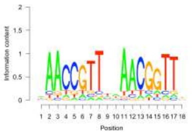	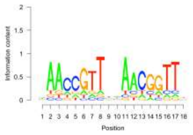
Observed number motifs	38	5	46	430	2982
Expected number motifs	0,02	0.78	15	182	1504

Supplementary Table 8. Number of sites overlapping predicted coding sequences.

Predicted CDS cover about 87.8% of the *C. abyssi* genomes (4.37 Mb out of 4.98 Mb).Therefore, about 88% of sites in each category are expected to overlap with a predicted CDS in at least one position.

	0-mismatches	1-mismatch	2-mismatches	3-mismatches	4-mismatches
Total number of motifs	38	5	46	430	2982
Observed number of motifs overlaping predicted CDS	13	1	44	390	2714
Expected number of motifs overlaping predicted CDS	33	4	40	378	2619

Supplementary Table 9. Genes overlapping with sites with zero mismatches in the core region.

Feature id	start	length	strand	product	Best BLAST hit
Caby_0174	202530	87	-	hypothetical protein	ND
Caby_0802	929560	82	-	hypothetical protein	ND
Caby_1033	1218577	44	-	hypothetical protein	ND
Caby_1034	1218888	43	-	hypothetical protein	ND
Caby_1035	1219229	40	-	hypothetical protein	ND
Caby_1036	1219540	41	-	hypothetical protein	ND
Caby_1404	1699975	50	-	hypothetical protein	ND
Caby_2417	2878653	41	-	hypothetical protein	ND
Caby_2444	2903827	45	+	hypothetical protein	ND

Caby_2445	2904207	39	-	hypothetical protein	ND
Caby_3268	3890323	42	+	hypothetical protein	ND
Caby_3333	3962353	73	+	hypothetical protein	ND
Caby_3835	4538387	46	+	hypothetical protein	ND

Supplementary Table 10. Number of sites with various types of intergenic region (between convergent, divergent and co-directed genes)

	**Convergent**	**Divergent**	**Co-directed**
Number of intervals in genome (length>18 nucleotides)	388	433	1995
0-mismatch sites	9	1	15
1-mismatch sites	0	2	2

Supplementary Table 11. Directionality of sites: the distribution of 0 and 1 mismatch sites by two central nucleotides along the genome.

The exact binomial test was used to detect over-representation of sites with the given dinucleotides in the center in either half of the genome. The p-values are provided for the one-tailed exact binomial test. The success probabilities for the test are taken from the actual distribution for the dinucleotides of interest in the two corresponding halves of the genome (0.51 of all "GA"+"AA"dinucleotides located in the first half and 0.52 of all "TC"+"TT" in the second half). The results may indicate strand preference in the distribution of sites.

	**Motifs with central "GA" and "AA" on the first half of the genome (from origin)**	**Motifs with central "TC" and "TT" on the second half of the genome (from origin)**
0 mismatch	19 of 25 (p-value=0.01)	10 of 12 (p-value=0.03)
0-1 mismatch	23 of 29 (p-value=0.002)	11 of 13 (p-value=0.017)
Expected frequencies	0.51	0.52

Supplementary Table 12. Putative nickel responsive regulator NikR binding site.

The genome of *Caldithrix abyssi* LF13 was scanned with position-specific weight matrix (PSWM) of NikR binding site from order *Desulfuromonadales* (Proteobacteria/delta), retrieved from RegPrecise website (http://regprecise.lbl.gov/RegPrecise/sites.jsp?regulog_id=1720). This order was selected based on sequence similarity of the corresponding transcription factors and availability of manually curated regulatory motifs.

**Feature id**	**Position to start**	**motif**	**logo**
Caby_1316)	-37	**GTGTTAC** ATTGA **TGA** AAAAA **GTAACA**	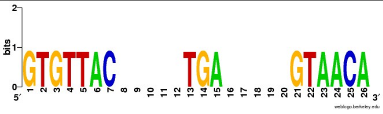
Caby_2622	-123	**GTGTTAC** TCATG **TGA** TTTTC **GTAACA**	

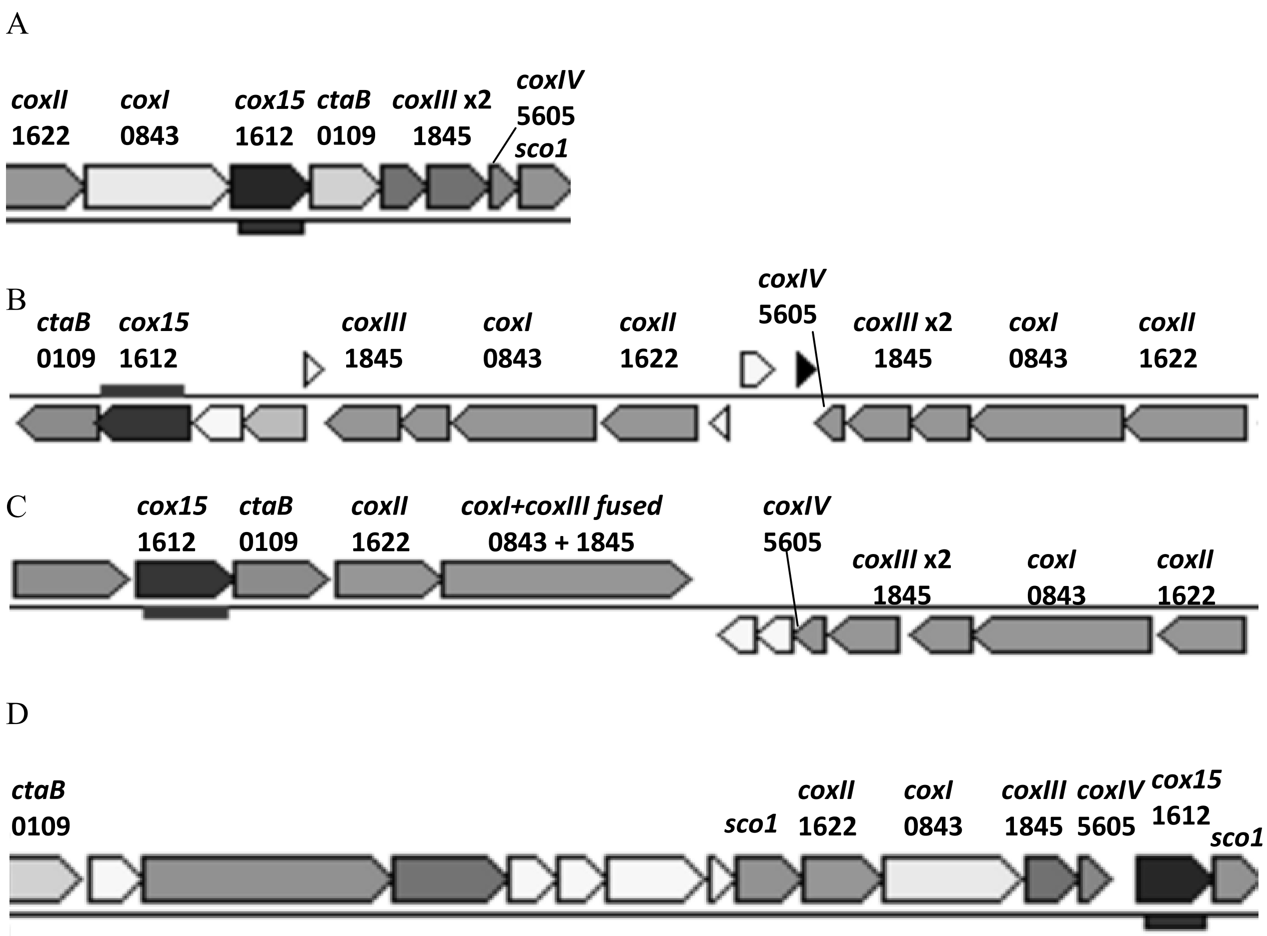

Supplementary Figure 1. Organization of the cytochrome *c* oxidase chromosomal cassettes, containing COGs 0843, 1622, 1845, 5605, 1612, 0109. A – "conservative" cassette revealed in the majority of aerobic strains (106 of 109) in which *coxIII* gene is duplicated, and the genes of heme-processing enzymes – heme *a* synthase (Cox15) and polyprenyltransferase (CtaB) – are clustered together and encoded between *coxI* and *coxIII*, right downstream of heme-containing catalytic subunit CoxI; B and C – "atypical" cassettes of a halophilic aerobe *H. anticariensis* and a thermophilic aerobe *M. chliarophilus*, respectively, sharing core features with the A-type cassette but having different positions of the Cox15-CtaB subcluster; D – chromosomal cassette of the anaerobe *Caldithrix abyssi*. Underlined is the gene of heme *a* synthase *cox15* from the subcluster of heme processing genes. Indicated over the drawings are gene names and corresponding COG term numbers, *sco1* – the gene of a copper chaperone.

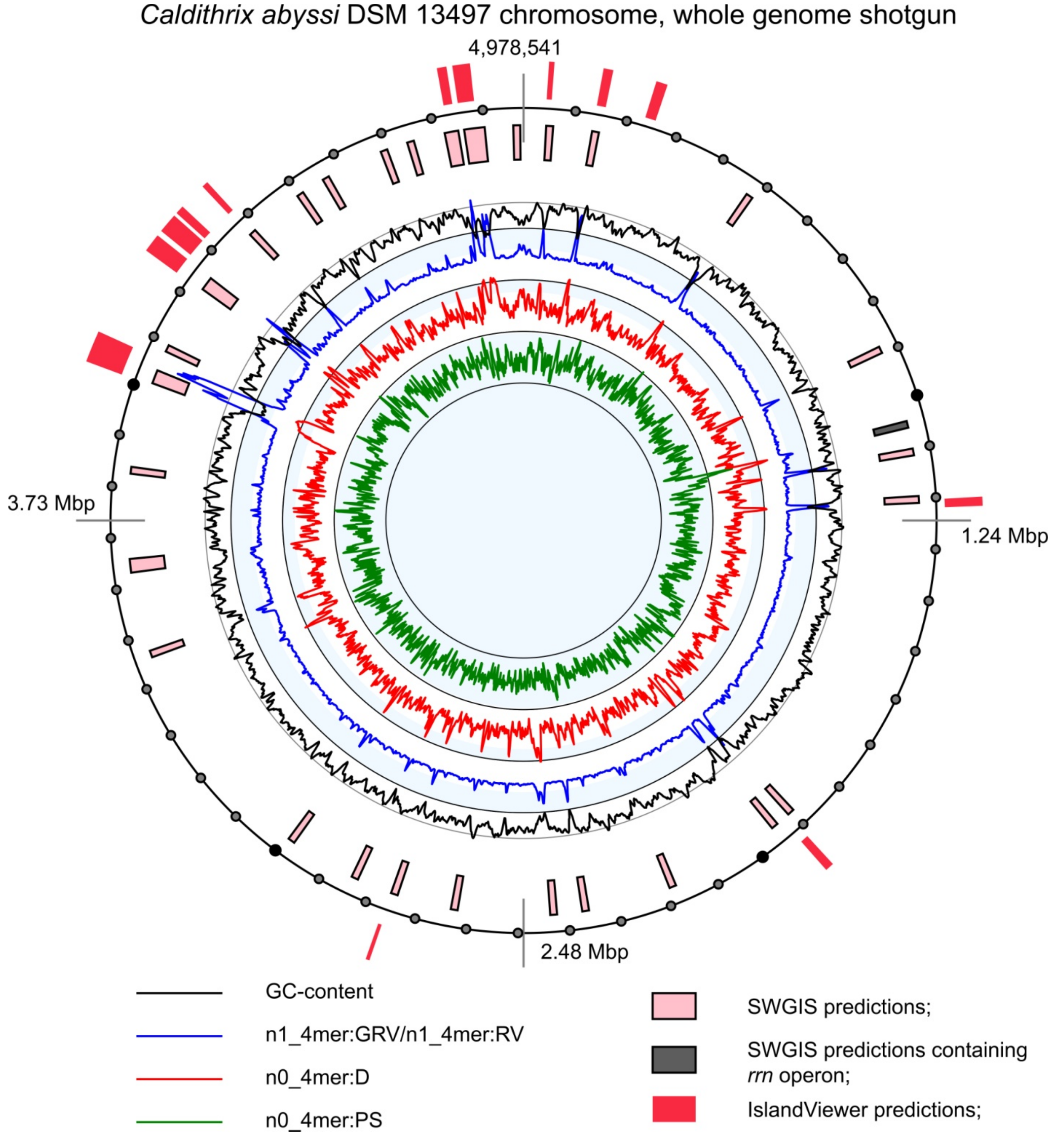

Supplementary Figure 2. *Caldithrix abyssi* genome map with genome islands predictions.

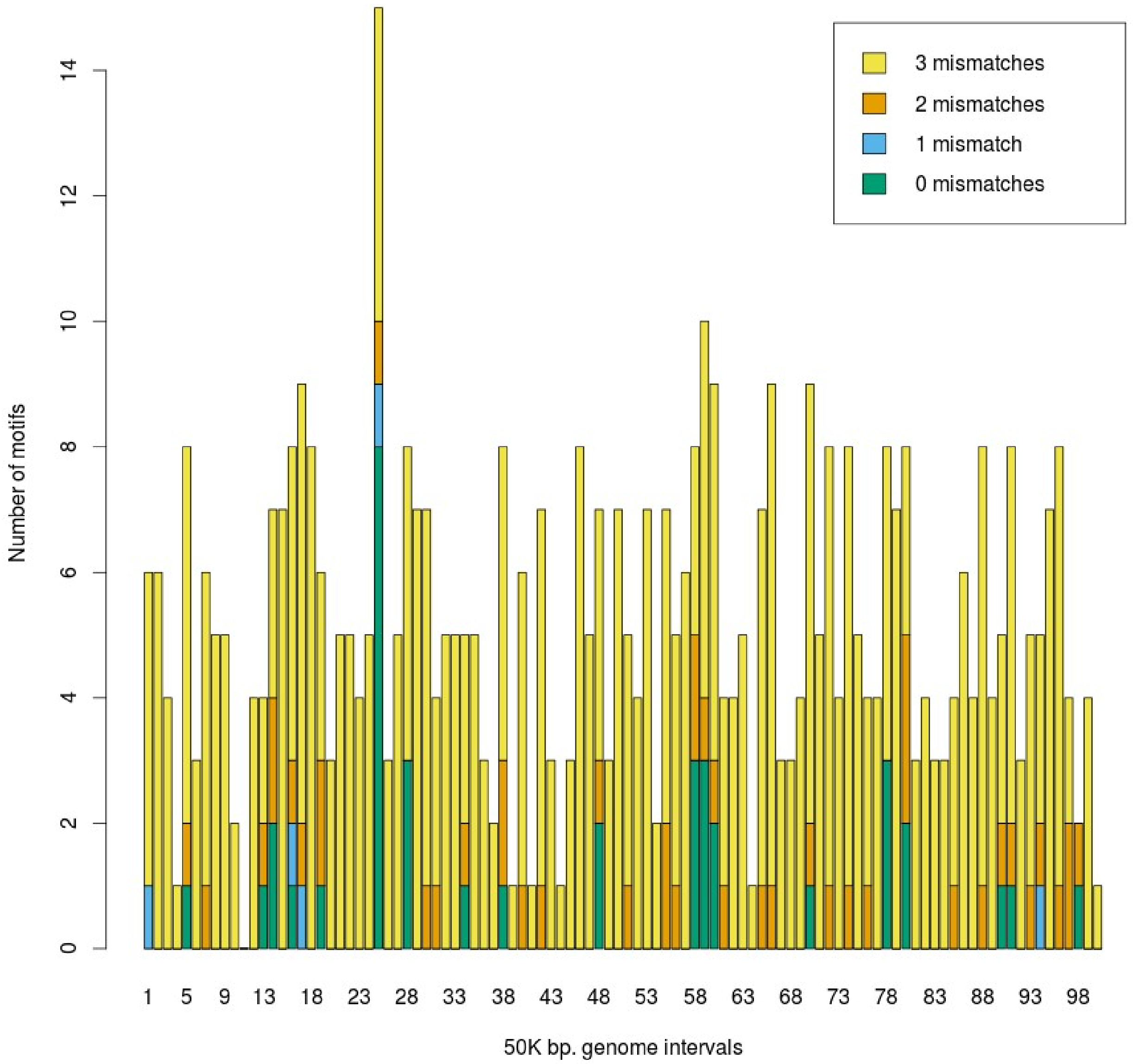

Supplementary Figure 3. Distribution of sites with different numbers of mismatches in the core region of conserved motif along the genome

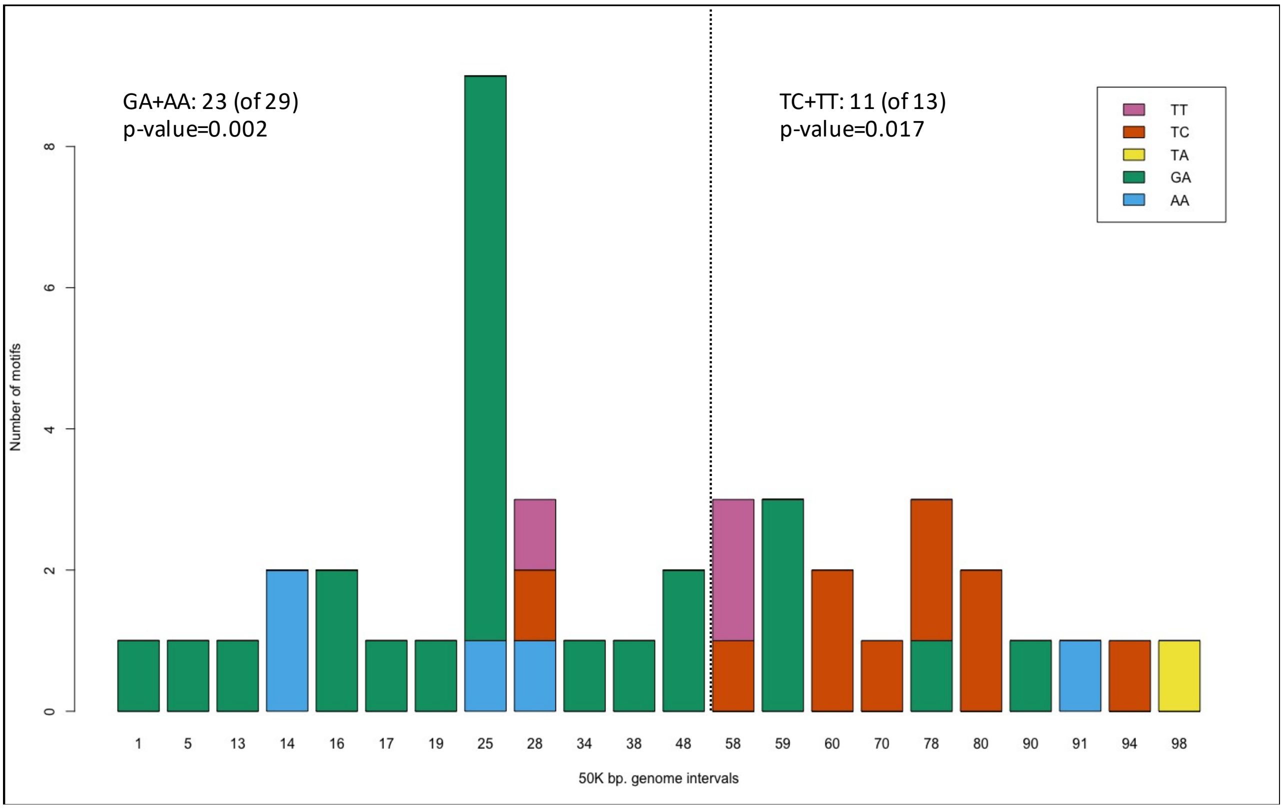

Supplementary Figure 4. Directionality of sites: the distribution of 0 and 1 mismatch sites by two central nucleotides along the genome. Genome coordinates start from the predicted origin of replication. The dotted line splits the genome into two halves at the expected terminus position.

```
>> AdoCbl_riboswitch  AdoCbl riboswitch
 rank     E-value  score  bias mdl mdl from   mdl to       seq from      seq to       acc trunc   gc
 ----   --------- ------ ----- --- -------- --------    ----------- -----------      ---- ----- ----
  (1) !   3.4e-05   32.9   1.1  cm        1      150 []     4790283     4790401 + .. 0.91    no 0.57

                                          vv                                                          NC
                             ::::::::::::((((((((((((,,,,,,<-<<<<_______>>>>>,,<<<___>>>,<<<---<-<~~~~~>->---->> CS
  AdoCbl_riboswitch       1 aauugAauAUAuAagaacuuaggaaAuGGAAGUCugGUGaaAAUCcaGCaCGGuCcGCCACUGUGAaaa*[9]*uuuAAGUCA 88
                            AA++GAA    U+::::  :       GGAAG ::GGUGAAAAUCC::CAC G:CCC:C ACUGUGA:+:      : :AAG CA
        Caldithrix 4790283 AAAAGAACCCUUUCAGUAAC------GGGAAGCGCGGUGAAAAUCCGCCACUGCCCCGCAACUGUGAUGG*[4]*CGAAAGCCA 4790359
                            ***************9877......79***************************************975..6..469****** PP

                                      vv               v                       v          NC
                            >,,,,,,))))))))))))------<<<<<<-<<<<<<_______>>>>>->->>>>>>:::  CS
  AdoCbl_riboswitch      89 GGUCuUUuuccuaaguucuUuaAguagccauaccgCuUCGAGGcAAaGcguggauggcuaUu  151
                            G  C         :  ::::U   + :: :: +C        GG A     UGG :: :: U+
        Caldithrix 4790360 GAACAAC-----GCCACUGU--UCCGCUUUAGC--------GGGA-----UGGGAAGGCCUG  4790402
                            **99999.....7789****..999**999999........5666.....377699******  PP
```

B

```
>> TPP  TPP riboswitch (THI element)
 rank     E-value  score  bias mdl mdl from   mdl to       seq from      seq to       acc trunc   gc
 ----   --------- ------ ----- --- -------- --------    ----------- -----------      ---- ----- ----
  (1) !   9.7e-18   74.0   1.0  cm        1      105 []     4720261     4720161 - .. 0.98    no 0.51

                       v                                     vv          vv                                NC
                  :::::[[[[[((((,,<<<<<___......__>>>>>,,,,,<<<<<<_>>>>>>,,)))),,,,,<<<<--<<<<______>>>>-->>> CS
        TPP     1 aaaaaccacuagGGGuGCcccaaa......aagggGCUGAGAuggaagucuuccaaACCcuUuGAACCUgAUCcGGuUAAUACCgGCGuAG 85
                  A  A  C::U:GGGGUGC::C+         AG::GCUGAGA               +ACCC:UUGAACCU:AUC:GG UAAUACC:GCG:AG
  Caldithrix 4720261 AGCAGUCCGUGGGGGUGCUUCUGGuaaaacCAGAGGCUGAGAGC----------AGACCCCUUGAACCUGAUCUGGGUAAUACCAGCGCAG 472018[illegible]
                  ********************99999999989**********97..........7************************************ PP

                     v             NC
                  >,,]]]]]:::::::::::: CS
        TPP    86 GGAaguggaaaaaaaaaaau 105
                  G AA::G AAAAAAAA+AA
  Caldithrix 4720180 GAAACGGCAAAAAAAAUAAC 4720161
                  ******************** PP
```

C

```
>> MOCO_RNA_motif  Moco (molybdenum cofactor) riboswitch
 rank     E-value  score  bias mdl mdl from   mdl to       seq from      seq to       acc trunc   gc
 ----   --------- ------ ----- --- -------- --------    ----------- -----------      ---- ----- ----
  (1) !   2.6e-19   79.5   0.2  cm        1      136 []     2401225     2401351 + .. 0.95    no 0.50

                                  v         vvv                                   vvv                          NC
                            ::((-(----((((((,,<<<<<<<<<<<---<<<<..____....>>>>-->>>>>>>>>>>,<<<<<______>>>>>,,<<<<< CS
  MOCO_RNA_motif          1 aaggaaaaaaaaCuCCGAGCcugugcaCCUAAgucg..aaaa....cgauAuGGugcacagGCCguggcaaaauagccaccAGGGug 81
                            A+::A:AA+ A CUCCGAGCC+  G::CCUA GUC:  + A     :GAUAUGG::C   GGCC          A      AGGGU:
      Caldithrix 2401225 AUUAACAAUCAACUCCGAGCCAAAGAGCCUACGUCCugUGA-aucgGGAUAUGGUUCGC-GGCC---------GA-----UAGGGUA 2401295
                            ***********************************95444.5899*********9976.****.........67.....******* PP

                                v      v                            v                NC
                            <<<<<<____>>>>>>>>>>>><<_______>>,,,)))).))----)-))::::::: CS
  MOCO_RNA_motif         82 uagcugGAAAcggcuacgCCUCCCgUauUUGGAAAGGaG.uuaaauuaccaaaaau 136
                            :::C GGAAAC G::::GCCUCCCGU UUUGGAAAGGAG  UAAAU: ::A   AU
      Caldithrix 2401296 AGACGGGAAACGGUCUUGCCUCCCGUGUUUGGAAAGGAGcGUAAAUGGAAAUGGAU 2401351
                            ***************************************54899999********* PP
```

Supplementary Figure 5. Prediction of riboswitches: output of Infernal program.
